# Molecular Evolution of the NLR Gene Family Reveals Diverse Innate Immune Strategies in Bats

**DOI:** 10.3390/biom15121715

**Published:** 2025-12-10

**Authors:** Gang Liu, Fujie Han, Xinya Guo, Liya Yang, Nishan Du, Xue Zhao, Chen Zhang, Jie Peng, Kangkang Zhang, Jiang Feng, Ying Liu

**Affiliations:** 1 Jilin Provincial Key Laboratory of Animal Resource Conservation and Utilization, Northeast Normal University, Changchun 130117, China; liug219@nenu.edu.cn (G.L.); hanfj469@nenu.edu.cn (F.H.); zhangc067@nenu.edu.cn (C.Z.); pengj489@nenu.edu.cn (J.P.); zhangkk307@nenu.edu.cn (K.Z.); fengj@nenu.edu.cn (J.F.); 2 School of Environmental Sciences, Northeast Normal University, Changchun 130117, China; guoxy@nenu.edu.cn (X.G.); yangliya119@nenu.edu.cn (L.Y.); nishandu916@nenu.edu.cn (N.D.); 3 State Key Laboratory for Zoonotic Diseases, Key Laboratory for Zoonosis Research of the Ministry of Education, Institute of Zoonosis, and College of Veterinary Medicine, Jilin University, Changchun 130062, China; xuezhao23@mails.jlu.edu.cn; 4 Jilin Provincial International Cooperation Key Laboratory for Biological Control of Agricultural Pests, Jilin Agricultura University, Changchun 130117, China; 5 Key Laboratory of Vegetation Ecology, School of Environment, Institute of Grassland Science, Northeast Normal University, Ministry of Education, Changchun 130117, China

**Keywords:** chiroptera, evolution, NLR gene family, protein, immunity

## Abstract

Bats, as the world’s second-largest mammalian order, have garnered significant attention for their ability to harbor numerous viruses without exhibiting disease symptoms. Nucleotide-binding domain and leucine-rich repeat-containing receptors (NLRs) are crucial components of the immune system. This study conducted an evolutionary analysis of the NLR gene family across 26 bat species to investigate the molecular mechanisms underlying their role in viral resistance under high viral load pressure. We identified gene duplication events in multiple genes. The NLR gene family exhibited high conservation throughout evolution, which may contribute to the occurrence of gene duplication. This conserved genomic structure also ensures functional stability, safeguarding bats’ antiviral resistance. Most NLR genes primarily function within the type I interferon (IFN) signaling pathway and the NF-κB signaling pathway. The NLR gene family enhances the innate immune capacity of bats through the adaptive evolution of some genes, combining enhanced gene functionality with the maintenance of gene conservation at a low evolutionary rate. Moreover, bats employ diverse innate immune strategies, where multiple immune pathways collectively establish the innate immune barrier. The molecular evolution of this gene family provides new insights into the molecular mechanisms and functional pathways involved in the innate immune response of bats.

## 1. Introduction

Innate immunity serves as the first line of defense of the immune system and plays a vital role in immune responses. The innate immune system employs a set of germline-encoded receptors known as Pattern Recognition Receptors (PRRs), with innate immunity being intimately linked to PRR function. Among PRRs, Nucleotide-binding Oligomerization Domain (NOD)-Like Receptors (NLRs) constitute a specialized group of intracellular receptors. They detect pathogen-derived products with specificity, recognize Pathogen-Associated Molecular Patterns (PAMPs) and Damage-Associated Molecular Patterns (DAMPs), and initiate a broad sequence of defensive signaling pathways to eliminate harmful pathogens. NLRs have thus become essential components of the innate immune response [1,2,3,4,5,6]. Since Charles Janeway originally postulated the pattern recognition theory, several PRR families have been identified. These include membrane-bound receptors such as Toll-like receptors (TLRs), C-type lectin receptors (CLRs), and scavenger receptors (SRs), alongside cytosolic receptors including NLRs, Absent in Melanoma 2-like receptors (ALRs), RIG-I-like receptors (RLRs), Pyrin, and the cyclic GMP-AMP synthase –stimulator of interferon genes (cGAS–STING) pathway. The ligands recognized by these receptors have subsequently become an intensive area of research [7,8]. Among these PRRs, NLRs represent the most extensive and diverse family, both in terms of structure and function, as well as in their repertoire of recognized signals [9]. The NLR gene family is characterized by shared structural features: a variable N-terminal effector domain, a central Nucleotide-Binding Oligomerization Domain (NOD), and a C-terminal region composed of a variable number of leucine-rich repeats (LRRs). These domains are functionally distinct: the effector domain mediates protein–protein interactions and signal transduction, the NOD governs NLR activation and inflammatory responses, and the LRR region facilitates ligand recognition and binding [3,4,5,9]. These three structural domains collectively enable NLRs to evolve the capacity to recognize a broad repertoire of ligands, thereby initiating diverse functions through the evolution of these domains [9,10]. Based on phylogenetic relationships, NLRs can be further classified into three subfamilies: the NOD subfamily: comprising *NOD1*, *NOD2*, *NOD3/NLRC3*, *NOD4/NLRC5*, *NOD5/NLRX1*, and the Major Histocompatibility Complex (MHC) class II transactivator (*CIITA*); the NLRP subfamily (also known as NALPs): encompassing *NLRP1* to *NLRP14*; the Interleukin-1β Producing Activating Flagellin Sensor (IPAF) subfamily: including the ICE-protease activating factor (*IPAF/NLRC4*) and neuronal apoptosis inhibitory protein (*NAIP*) [11]. NLRs provide protection against infections caused by bacteria, viruses, fungi, and parasites [12]. Recognition of microbial products and exogenous danger signals promotes innate immune responses, thereby modulating inflammation, participating in cell death, and contributing to embryonic development; The NLR gene family plays a critical role in crop breeding and viral management [13,14,15,16]. Currently, extensive studies have been conducted on the NLR gene family across multiple plant groups [17,18,19,20,21]. In contrast, most animal studies focus on individual or limited genes. No studies have conducted a comprehensive screening and identification of the NLR gene family in bats based on genomic data. Only a limited number of studies have investigated parts of the NLR gene in bats. From now on, only the *NLRP1*, *NLRP3* and *NLRC5* genes have been studied in bats [22,23,24]. For example, *NLRP3* and *NLRC5* genes have been identified in the transcriptome of *Pteropus alecto* to be associated with antiviral immunity [24]. A comparative genomics study involving 19 bat species and 7 other mammals indicated that *NLRP1* is present in 13 bat species and all the 7 mammals. However, *NLRP1* is absent in 6 pteropodids species. This is due to the homologous Long interspersed nuclear element-1 (LINE-1) insertion. The absence of this gene may enable bats to reduce the inflammatory response to viruses [23]. Simultaneously occurring is the duplication of the *Complement Component 5a Receptor 2 (C5AR2)* gene. This gene encodes the receptor for the complement anaphylatoxin C5a, which regulates *NLRP3* inflammasome activation and modulates the anti-inflammatory function of IL-1β release by macrophages [23,25,26,27]. The phenomenon of NLR gene duplication is highly prevalent. The *NLRP1*, *NLRP4*, and *NLRP9* genes exhibit specific duplication patterns in rodents [28]. Positive selection-driven evolutionary mechanisms suggest that the duplication of these *NLRP* genes enhances the ability of mice to adapt to new environments [29,30]. The NLR gene family also plays a role in various physiological activities in other mammals. For example, multiple studies demonstrate that *NLRP3*, as a critical inflammasome component, plays essential roles in diverse conditions such as autoinflammatory and metabolic disorders [31,32,33]. The *NLRP1* inflammasome has been implicated in multiple dermatological conditions [34,35]. *NLRP6* exerts significant roles in inflammation suppression and mitigation of hepatic disorders, among other functions [36]. *NLRX1* acts as a mitochondrial signaling adaptor that potentiates virally triggered autophagic clearance [37,38]. In contrast to *NLRC3* which augments antiviral T-cell immunity yet constrains pathological inflammatory responses to infection [39,40]. Concurrently, the NLR gene family plays critical roles in reproductive processes: both *NLRP5* and *NLRP7* function during embryonic development regulation [41,42,43]. *NLRP9* is specifically expressed in the ovaries during early embryonic development and exhibits anti-rotaviral clearance function [44,45,46,47]. Collectively, the NLR gene family plays critical roles in development, reproduction, and immunity across organisms.

Bats represent the second most diverse mammalian order globally, comprising over 1500 species [48]. Their unique biological, ecological, immunological, and genetic features enable them to harbor a greater diversity of viruses and bacteria than most other mammals [49,50,51,52]. Their flight capability facilitates wider dissemination of viruses and bacteria. Notably, these pathogens—primarily RNA viruses—are highly pathogenic in humans and frequently associated with aberrant innate immune activation [53,54,55,56]. Upon viral challenge, bats exhibit attenuated inflammatory responses compared to other mammals infected with identical viral strains [57,58,59,60]. The Coronavirus Disease 2019 (COVID-19) pandemic has accelerated research focus on bats as reservoir hosts, driving increased investigation into viral transmission dynamics and host–virus receptor interactions [49,61,62,63,64,65,66]. Concurrently, research targeting bat-specific immune genes has expanded significantly, particularly focusing on molecular adaptations enabling viral tolerance [67,68,69].

PRRs constitute specialized receptors representing critical components of the host innate immune system. Their evolutionary trajectories have garnered substantial scientific interest due to potential selective pressures exerted by diverse co-evolving pathogens [67,70]. Bats’ capacity to limit immunopathology during viral infections likely stems from evolutionary adaptations in their immune system, which may contribute to positive selection acting on associated immune genes [70]. The NLR gene family, which serves critical roles in infectious diseases, autoimmune disorders, and autoinflammatory conditions, has been extensively studied in various plant taxa [71,72,73,74]. While numerous investigations confirm the essential functions of NLR members in animal immunity, comprehensive evolutionary analyses of this gene family across mammalian lineages remain scarce. As major pathogen reservoirs, bats harbor diverse viruses and bacteria yet exhibit exceptional disease resistance. Immune genes are fundamentally implicated in this phenomenon. Under intense pathogen pressure, bat immune genes likely experience heightened selective constraints. Consequently, bats exhibit the most pronounced signatures of positive selection in immune system-related genes among mammals—a testament to their evolutionary adaptations for viral coexistence [70].

As pivotal components of the immune system, the evolutionary dynamics of the NLR gene family significantly influence bat immunocompetence. Investigating NLR gene evolution in bats provides critical insights into their functional roles in antiviral immunity and establishes a theoretical foundation for understanding bat immune adaptations. Utilizing available bat genome assemblies, we conducted an analysis of the molecular evolutionary patterns within the NLR gene family across diverse bat species. This study aimed to elucidate the molecular mechanisms underpinning the antiviral functions of bats, thereby providing theoretical support for subsequent research on bat immunity and antiviral drug development.

## 2. Materials and Methods

### 2.1. Genome Data Collection and Sequence Alignment

We collected and organized the genomes of bat species available in the National Center for Biotechnology Information (NCBI) database, selecting a total of 26 Chiroptera species genomes (Table S1), including: Vespertilionidae (n = 8); Phyllostomatidae (n = 3); Emballonuridae (n = 2); Pteropodidae (n = 2); Hipposideridae (n = 4); Rhinolophidae (n = 7); The NCBI serves as an invaluable resource for investigating species evolution using available molecular data, enabling rapid elucidation of biological phenomena. Details of selected chiropteran species and their corresponding genomic assemblies are provided in Table S1. NLR gene identification was performed through online BLAST (2.17.0) searches and NCBI database queries. Protein-coding sequences of NLR genes from sequenced chiropteran species were retrieved from NCBI and utilized as query sequences to screen for NLR gene family sequences in other target genomes. For each target genome, a local database was constructed. BLASTN algorithm [75] implementations within BLASTn (TBtools v2.038) [76] were employed to search these databases using NLR coding sequences. Candidate sequences were validated as significant genome matches based on an E-value cutoff of 1 × 10^−5^, selecting high-scoring segment pairs (HSPs). Target sequences were subsequently obtained using GeneWise (European Bioinformatics Institute, Cambridgeshire, UK; https://www.ebi.ac.uk/Tools/psa/genewise/, accessed on 7 December 2025). Finally, all retrieved sequences were analyzed in MEGA X (v7.0.14) [77] to detect premature termination codons, which were excised from the final dataset.

### 2.2. Molecular Evolution Analysis

Phylogenetic analysis was performed on the identified NLR gene family sequences. MEGA X (v7.0.14) was used to convert the obtained target nucleotide sequences into amino acid sequences. Multiple sequence alignment of the translated amino acid sequences was conducted using MUSCLE (v3.8.31) with the Log-Expectation (LE) algorithm [78]. Phylogenetic trees were constructed under the maximum likelihood framework using IQ-TREE [79] with 1000 bootstrap replicates. We used distantly related canids as outgroups to anchor this phylogenetic tree. Our outgroup sequences were derived from genomic identification results of *Canis lupus familiaris*. Using canine sequences as comparators or outgroups has been established practice in multiple published phylogenetic studies of gene families or immune-related genes [80,81]. The optimal substitution model was determined using ModelTest and the Bayesian Information Criterion (BIC) [82], with GTR + T identified as the best-fit model. Final trees were visualized and annotated in the Interactive Tree of Life (iTOL) platform [83]. Positive selection pressure was assessed by calculating the ratio (ω) of non-synonymous (dN) to synonymous (dS) substitutions in homologous protein-coding sequences, where ω > 1, ω < 1, and ω = 1 indicate positive selection, purifying selection, and neutral evolution, respectively [84]. Based on codon alignments and a TimeTree-derived species phylogeny (http://www.timetree.org/, accessed on 7 December 2025), selection analyses were performed on chiropteran NLR genes using PAML v4.9 [85]. Branch-specific selection analyses were subsequently conducted for each bat family under identical selection parameters, with likelihood ratio tests (LRTs) subjected to multiple testing correction [86]. Due to the presence of pseudogenes and limitations in species coverage, NLR genes from certain families cannot be predicted using the site model. Therefore, the results will lack the outcomes for individual site models. Fortunately, this does not invalidate the experimental conclusions. The primary reason is that pseudogenes, due to premature termination, are typically subject only to neutral drift and do not undergo genuine adaptive positive selection [87].

Codon-based site models in CodeML [88] were employed to identify signatures of natural selection in chiropteran NLR genes by detecting variable evolutionary rates and functional divergence across amino acid sites. These models permit heterogeneous ω ratios among codon positions within a sequence. Positive selection sites were tested using nested model comparisons: the null model M7 (beta distribution, 0 < ω < 1) versus the alternative model M8 (beta distribution + ω > 1 class). Model acceptance was determined by likelihood ratio tests (LRTs). When LRT *p*-values indicated statistical significance (*p* < 0.05), the alternative M8 model (positive selection model) was selected. For significant models, Bayes Empirical Bayes (BEB) analysis identified positively selected sites with *p* > 0.9 [89]. Positive selection results were validated using the Datamonkey platform’s multiple algorithms (SLAC, FEL, and REL) (http://www.datamonkey.org/, accessed on 7 December 2025) [90].

Branch models were implemented to assess lineage-specific evolutionary patterns of NLR genes, that is, adaptive evolution across different bat families. This involved comparing a positive selection model (MA: model = 2, NSsites = 2) against a null model (Fix_ω = 1, ω = 1). The MA model allows independent ω ratios for each branch in the phylogeny, whereas the null model constrains all branches to share a single ω value of 1 [91].

To determine whether positive selection targets specific sites on foreground branches, branch-site models were implemented with each bat family designated as the foreground branch. Positive selection acting on specific codons was tested by comparing two models. Null model: Fix_omega = 1 (ω = 1 for all sites/branches), Alternative model: MA (model = 2, NSsites = 2; allows ω > 1 on foreground branches) [92]. For significant models (LRT *p* < 0.05), an empirical Bayesian approach calculated posterior probabilities of positively selected sites. Sites with *p* > 0.9 were considered under positive selection.

### 2.3. Protein Three-Dimensional Structure Prediction

Potential positive selection sites were identified by at least two machine learning (ML) methods. To determine whether NLR gene positive selection sites localize within functional protein domains, we designated Rhinolophus genomes (selected for absence of pseudogenes) as reference sequences. Functional domains of all NLRs were annotated using SMART [93] (http://smart.embl-heidelberg.de/, accessed on 7 December 2025) website to confirm the functional domains of all NLRs genes; Subsequently, protein tertiary structures were predicted via I-TASSER [94] (https://aideepmed.com/I-TASSER/, accessed on 7 December 2025). Finally, candidate positive selection sites were mapped onto predicted structures using PyMOL (3.1).

### 2.4. Collinearity Analysis

Using species genomes downloaded from NCBI, we selected *Rhinolophus sinicus* and *Rhinolophus ferrumequinum* for synteny analysis. The entire analysis and visualization process were performed by using TBtools (v2.038) [76].

## 3. Results

### 3.1. NLR Gene Identification and Gene Tree Reconstruction

**Figure 1:**
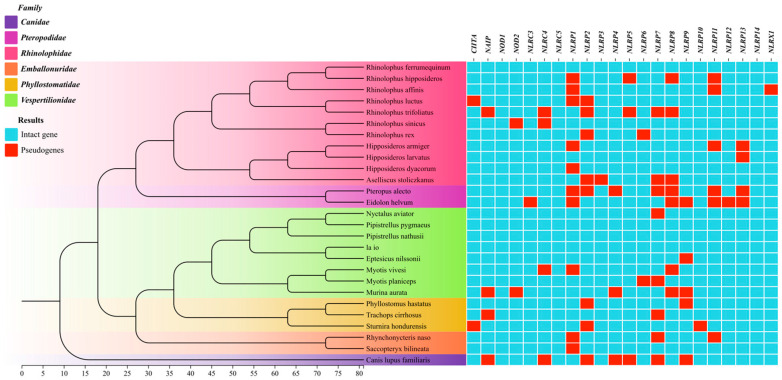
Species tree for the animals used in this study and the intact NLR genes number in these animals. (The species tree was constructed based on information from the Timetree website. *C. lupus familiaris* as outgroups in the species tree.).

**Figure 2:**
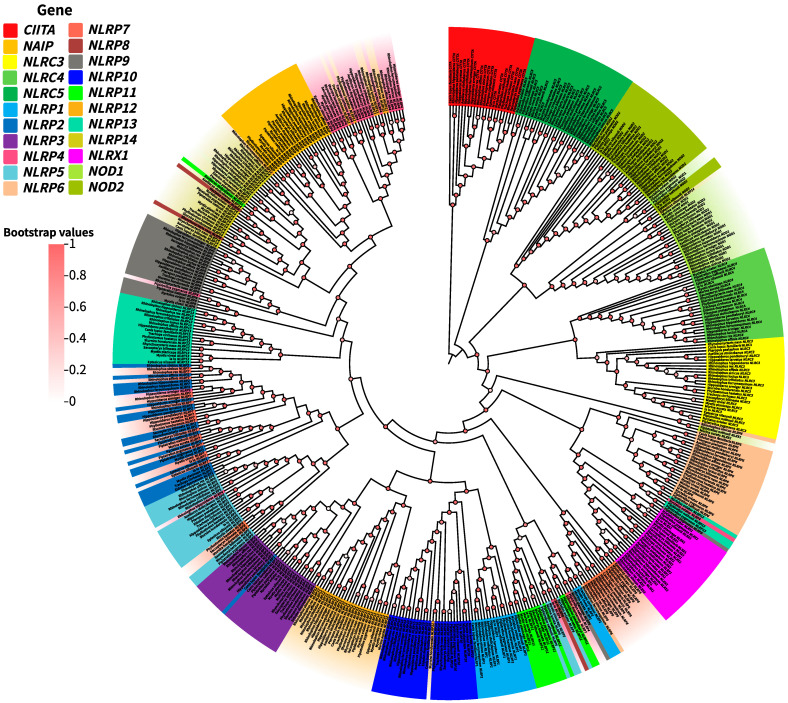
Phylogenetic tree of the NLR genes in the Chiroptera. (Phylogenetic trees are constructed based on the amino acid sequences of all genes. The color intensity at each branch node represents the confidence level.).

We identified and characterized NLR genes across 26 chiropteran species, yielding 572 NLR loci comprising 501 intact genes and 71 pseudogenes. Canine NLR genes (*Canis lupus familiaris*) were additionally characterized as an outgroup. Figure 1 shows the phylogenetic tree of the selected species downloaded from Timetree. Different colors are added according to the different families to which the species belong, and the results of gene screening are combined. A maximum-likelihood phylogeny constructed from all NLR genes (Figure 2) revealed topological congruence with the species tree. Fifteen NLR subtypes—*CIITA*, *NAIP*, *NOD1*, *NOD2*, *NLRC3*, *NLRC4*, *NLRC5*, *NLRX1*, *NLRP3*, *NLRP6*, *NLRP8*, *NLRP9*, *NLRP10*, *NLRP12*, and *NLRP13*—formed distinct monophyletic clusters with minimal inter-clade associations. Notably: *NLRP2* and *NLRP7* clustered across Chiroptera. *NLRP11* and *NLRP4* co-clustered in Rhinolophidae and Phyllostomidae. *NLRP1*, *NLRP4*, *NLRP5*, and *NLRP11* formed a Vespertilionidae specific clade (Figure 2).

### 3.2. Evolutionary Selection Characteristics of NLR Genes

**Figure 3:**
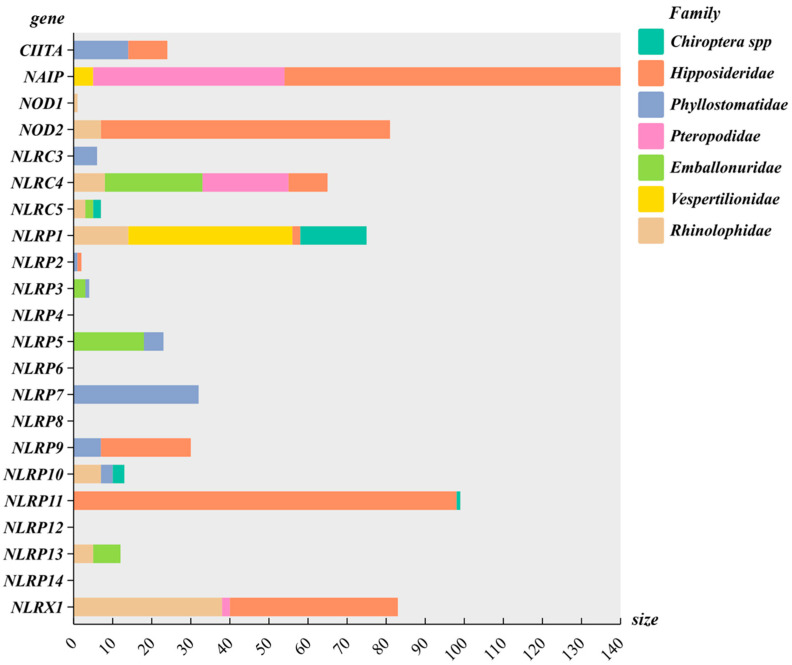
Number of positively selected sites for genes in the NLR gene family. (The horizontal axis represents the number of positively selected sites.).

Site model analyses identified positively selected sites within each NLR gene, indicating adaptive evolution under positive selection. Our gene-based positive selection site analysis detected 697 positive selection sites (*p* > 0.95). The specific number of subjects for each category is shown in Figure 3. Positive selection was detected at 47 sites in Vespertilionidae, 47 in Rhinolophidae, 55 in Emballonuridae, 73 in Pteropodidae, 69 in Phyllostomidae, and 347 in Hipposideridae (Table S2).

Branch model analyses were employed to detect selection pressures on NLR genes across chiropteran lineages, identifying potential adaptive evolution in specific clades. The estimated ω ratios ranged from 0.0001 to 1.0916 (Table S3). We consider ω > background branches to indicate a significantly elevated evolutionary rate. Genes *NOD2*, *NLRC5*, *NLRP1*, *NLRP8* and *NLRX1* in Vespertilionidae exhibit higher evolutionary rates. The evolutionary rates of the *NOD2*, *NLRP5*, *NLRP8*, *NLRP11* and *NLRX1* genes in Phyllostomidae are higher than background branches. The evolutionary rates of the *NOD2*, *NLRC5*, *NLRP8*, *NLRP11* and *NLRX1* genes in Hipposideridae are higher than background branches. Genes *NLRC5*, *NLRP8*, *NLRP11* and *NLRX1* in Pteropodidae exhibit higher evolutionary rates. The evolutionary rates of the *NLRC*5 and *NLRP8* genes in Emballonuridae are higher than background branches. The evolutionary rates of the *NLRP1*, *NLRP5*, *NLRP8* and *NLRX1* genes in Rhinolophidae are higher than background branches. It is worth noting that while all listed genes showed elevated evolutionary rates, only *NLRP1* in Vespertilionidae exhibited signatures of positive selection (ω > 1). The remaining NLR genes across all families were under significant purifying selection (ω < 1) (Table S3).

To delineate lineage-specific evolutionary pressures on NLR genes, we implemented branch-site models with each bat family designated as the foreground branch and remaining taxa as background. We identified a large number of positively selected sites in *NLRP1* and *NLRP8* of the Vespertilionidae, *NLRP2*, *NLRP10* and *NLRX1* of the Hipposideridae, and *NLRC3* and *NLRP10* of the Emballonuridae; A small number of positive selection sites were identified in the *NOD1* gene of Phyllostomatidae and *NLRP1*, *NLRP10*, *NLRP13* and *NLRX1* in Rhinolophidae. No positively selected sites were identified in Pteropodidae (Table S4).

### 3.3. Prediction of the Three-Dimensional Structure of NLR Gene Proteins and Positive Selection Site Marking

**Figure 4:**
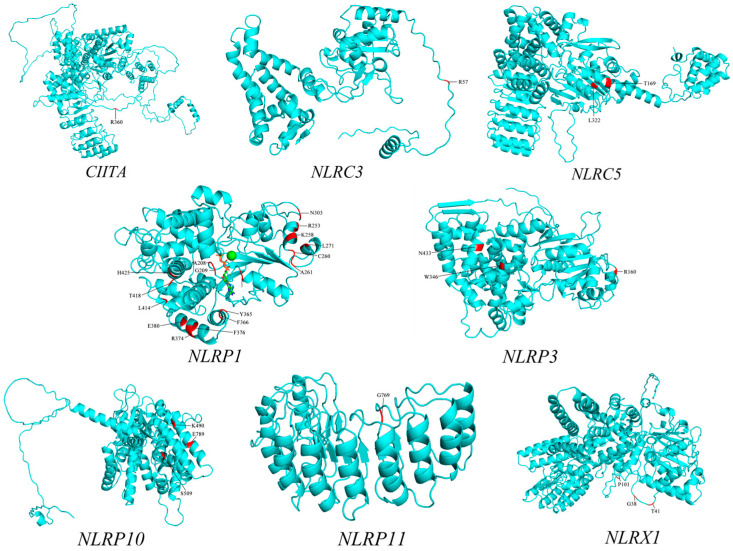
Prediction of the three-dimensional structure of NLRs gene proteins and calibration of positive selection sites. (Protein tertiary structures were predicted via I-TASSER. Candidate positive selection sites were mapped onto predicted structures using PyMOL.).

In order to intuitively reflect whether the positive selection site is located in the important structural functional domain of NLR proteins, we used the protein sequence of the NLR gene of *Rhinolophus ferrumequinum* as a reference and performed three-dimensional structure prediction of the protein using PyMOL software (Figure 4). The positively selected sites identified through screening were marked in the three-dimensional structure domain of the protein to further assess their functional significance.

### 3.4. NLR Gene Family Collinearity Analysis

**Figure 5:**
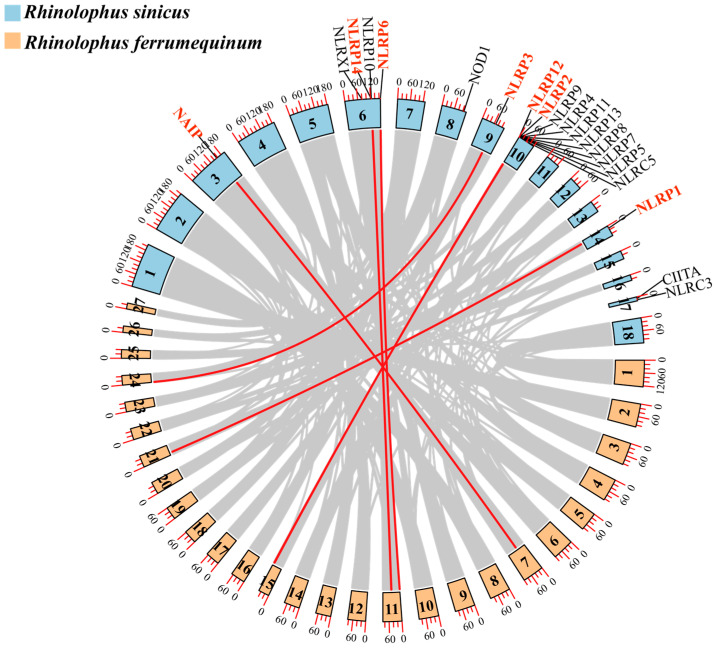
Gene family collinearity analysis. (The genes highlighted in red are those exhibiting colinearity.).

To further investigate the evolutionary relationships within the NLR gene family, we conducted a gene family colinearity analysis on two species from Rhinolophidae. Through collinearity analysis, we identified a total of seven genes exhibiting colinearity (Figure 5). These genes are mainly distributed across five chromosomes.

## 4. Discussion

Our comprehensive identification and analysis of NLR gene family members across Chiroptera revealed 572 NLR genes, comprising 501 intact genes and 71 pseudogenes. Abundant positively selected sites were detected in NLR genes across multiple bat species, with significant variation in both the identity of positively selected genes and the distribution of selected sites among lineages. This demonstrates divergent evolutionary trajectories of the NLR gene family within the order Chiroptera.

Bats, the only flying mammals, exhibit exceptionally high metabolic rates yet demonstrate remarkable longevity. They asymptomatically harbor numerous bacteria and viruses, including diverse potentially zoonotic viruses capable of cross-species transmission [53,54,55,56]. Pathogenicity of these viruses frequently correlates with dysregulated innate immune activation [57,58,59,60]. However, when bats are attacked by these viruses, even when high viral loads are detected in serum or tissue, there are no or very few signs of disease [67,70]. Pattern recognition receptors (PRRs) serve as the first line of defense in the innate immune system, triggering an immune response in the host by recognizing pathogen-associated molecular patterns (PAMPs) [95,96]. The emergence of pseudogenes was consistently associated with the appearance of stop codons, leading to premature termination of the gene. Gene deletion or pseudogenization can exert significant effects on various biological functions. During gene screening, we observed that certain species possess pseudogenes within their NLR gene family. Pseudogene formation may arise from multiple factors, including genome assembly quality, epigenetic modifications (e.g., DNA methylation), and the accumulation of mutations following gene duplication [97,98]. Notably, homologous LINE-1 insertions have been shown to cause *NLRP1* gene deletions in several bat species [23]. Phylogenetic analysis revealed significant differentiation within the NLR gene family, with multiple genes undergoing gene duplication events. The *NLRP7* gene underwent gene duplication, mainly involving the *NLRP2* gene, and this duplication phenomenon exists in all bats. The *NLRP11* gene in Rhinolophidae and Phyllostomidae has undergone duplication, with *NLRP4* as the main gene. In Vespertilionidae, gene duplication events primarily derived *NLRP1*, *NLRP4*, and *NLRP5* from an ancestral *NLRP11*. There are many possible causes for this phenomenon, such as potential duplications and deletions, gene conversions, or incorrect identification of genes that could lead to these results. Gene duplication plays a crucial role within gene families, enhancing the function and diversity of the immune system [99,100,101,102]. Multiple studies have demonstrated that gene duplication is prevalent within the gene family [103,104,105]. The emergence of pseudogenes within the NLR gene family may be associated with the accumulation of mutations following gene duplication. The observed gene-specific duplication patterns are likely associated with distinct ecological selective pressures and pathogen exposure across species [106,107].

As is well known, bats carry a large number of viruses but rarely develop diseases, which indicates that they possess a powerful immune system. NLRs play an important role in sensing virus pathogen-associated molecular patterns, and their inflammasome activation and pyroptosis are key biological mechanisms mediating the host immune response after virus exposure [108,109,110]. *NLRP2* is a component of the inflammasome. It upregulates the expression of proinflammatory, chemotactic, and pro-fibrotic mediators by regulating the activity of the transcription factor NF-κB [111,112]. It also interacts with TANK-Binding Kinase 1 *(TBK1)* and negatively regulates type I IFN signaling during viral infection [113]. *NLRP7* is associated with decidualization and macrophage differentiation [114]. Viruses, bacteria, or other triggers may be absorbed by macrophages and neutrophils through phagocytosis and endocytosis [115]. At the same time, research has also found that the *NLRP2* cluster contains *NLRP7*, indicates that *NLRP7* may have originated from the duplication of the *NLRP2/7* ancestor in primates [116]. *NLRP4* can negatively regulate Tumor Necrosis Factor (TNF) and IL-1β-induced NF-κB activation, and can also negatively regulate type I interferon signaling in response to dsRNA, DNA, or viral infection, and regulate autophagy in response to bacterial infection [117,118,119]. *NLRP11* interacts with Mitochondrial Antiviral Signaling protein (MAVS) after RNA virus infection, promoting apoptosis in virus-infected cells, while also regulating the formation of *NLRP3* [120,121]. Both genes are involved in the transduction of inflammatory signals. *NLRP5* regulates mitochondrial biogenesis and respiratory activity and is associated with embryonic reproduction [122,123,124]. Vespertilionidae is the largest family in the order Chiroptera [125]. Its diverse habitats and feeding habits mean that it faces a wide variety of viruses. The replication of related genes may be related to the complexity of the viruses it faces. For example, during summer when insects are abundant, *La io* preys on insects for food, while in autumn when food resources become scarce, it switches to hunting birds. Therefore, compared to other bats, *La io* faces the additional challenge of combating pathogens carried by birds [126,127,128]. Existing research has demonstrated that the viral communities carried by southern bats exhibit seasonal variation and are highly correlated with their feeding habits [129,130]. In summary, some NLR genes in bats may have undergone genes duplication due to viral pressure, functional overlap, and ancestral homology, thereby further enhancing the bats’ immunity.

Through branch model analysis, we found that the NLR gene family has evolved conservatively in bats (Table S3). Only the *NLRP1* gene in Vespertilionidae has un dergone positive selection, while the remaining genes have been under purifying selection pressure. *NLRP1* inflammasome is a key driver of the innate immune response following SARS-CoV-2 exposure [131,132]. Among Chiroptera, only the immune genes of Vespertilionidae have undergone positive selection, which may be related to the higher diversity of viruses encountered by bat species [130]. Despite the current limitations of genomic resources, we have selected a smaller number of species for some families. But like other similar studies, the results can still support the conclusion when the number of species is small. Several previous studies have demonstrated that branch-site models can yield robust and interpretable signals of lineage-specific adaptive evolution even when the number of species is limited, provided that orthology is strictly curated and phylogenetic topology is well supported [85,133,134,135]. Like other immune genes, the NLR gene family is relatively conserved in terms of evolution. The gene collinearity also confirms the accuracy of our gene selection. The collinearity of gene families also demonstrates the conserved nature of the NLR gene family in evolution. This often indicates that these genes are critical in terms of function or regulation [136,137]. At the same time, studies have shown that the 2′-5′Oligoadenylate synthetase (OAS) gene family of Chiroptera is far more conserved than other species of the Laurasiatherian Mammals [68]. The highly conserved evolution of immune genes in bats under extremely high viral pressure also demonstrates that gene conservation can better ensure functional stability [138].

The frequency of selection signals for immune genes is more common in bats than in other mammals [139]. Although NLR genes are relatively conserved during evolution, positive selection has occurred at some sites during the evolutionary process. A large number of positive selection sites were found in each family, and even in genes that did not produce positive selection sites, positive selection signals were present. Among the bat families analyzed, Rhinolophidae exhibited the second-highest number of positively selected sites (83 sites). Viral sequence detections revealed significant differences across families: Hipposideridae (14 species) yielded 2143 viral sequences, while Rhinolophidae (14 species) produced 3737 sequences—markedly fewer than the 8646 sequences detected in Vespertilionidae (21 species) (https://www.mgc.ac.cn/cgi-bin/DBatVir/main.cgi?func=stat, accessed on 7 December 2025). The relatively low viral load in individuals Hipposideridae and Rhinolophidae is closely related to their innate immune capabilities and dietary habits. Compared to Hipposideridae, coronaviruses are more readily detected in Rhinolophidae [140]. Research on coronaviruses has also largely focused on Rhinolophidae [55,141,142,143,144,145,146,147,148]. Recent studies have shown that key sites in the Interferon-Stimulated Gene 15 (*ISG15*) gene of Rhinolophidae and Hipposideridae have undergone mutations, resulting in these two species exhibiting stronger antiviral activity in their *ISG15* genes [70]. As a key gene among type I IFN-induced genes, *ISG15* gene plays a crucial role in the immune signaling pathway of IFN [149,150]. Research has shown that both the NLR gene family and the *ISG15* gene are highly expressed in the innate antiviral immune response of mice [151,152]. This study found a large number of positive selection sites in members of the NLR gene family in Rhinolophidae and Hipposideridae, indicating that the NLR gene family plays an important role in the innate immune process of bats. This also confirms that, compared to other bat species, Rhinolophidae and Hipposideridae may possess stronger antiviral capabilities, particularly necessitating increased attention to the role of Hipposideridae in viral transmission processes. At the same time, the emergence of a large number of positive selection sites also indicates that pathogenic microorganisms exert continuous selection pressure on the host’s immune system through constant reproduction, mutation, and evolution. This suggests that in the “arms race,” there may be a process of coevolution between the host and the virus to better defend against the threat of viruses and pathogens. That is, the adaptive evolution of any species may exert selective pressure on another species, and this interrelationship may drive their coevolution [153]. This result strongly supports the “Red Queen hypothesis” [154]. We found that the NLR gene family also evolved differently in different bats. In the Hipposideridae, positive selection sites were found in the *NOD1*, *NLRP2*, *NLRP10*, and *NLRX1* genes, and the number of positive selection sites in *NLRX1* was significantly higher than in other genes. Rhinolophidae also has the most sites of positive selection for the *NLRX1. NLRX1* negatively regulates MAVS-mediated *IRF3* activation and type I IFN induction during viral infection [155]. *NLRX1* can block STING-TBK-mediated antiviral responses in HIV-1 infection and support the early antiviral response by regulating IRF-1 abundance post-transcriptionally The *NLRX1* gene can increase autophagy and reduce inflammatory responses [156]. In the Emballonuridae, the number of sites of positive selection in the *NLRC3* gene is the highest. *NLRC3* regulates STING-TBK-mediated type I IFN signaling and NF-κB pathways to control cellular immune responses [40]. *NLRP1* gene has the highest number of positive selection sites in Vespertilionidae. This is also consistent with the results of the phylogenetic tree analysis, which show that the *NLRP1* gene in Vespertilionidae is the only gene in the bat NLR gene family that has undergone positive selection. The above results indicate that different bats have different specific strategies for resisting viral attacks. The presence of positive selection sites in the *NLRX1* gene of both Hipposideridae and Rhinolophidae also suggests that this gene may play an important role in the resistance of these two families to viruses. The evolutionary characteristics of multiple genes indicate that the NLR gene family in bats primarily regulates the type I IFN signaling pathway and the NF-κB signaling pathway to assist bats in resisting viruses, which also demonstrates that these two pathways are important pathways for bats to resist viruses.

Marking positive selection sites of some NLR gene families on protein three-dimensional structures. We found that not all positive selection sites are located within their functional domains (Table S3). Research indicates that some members of the NLR gene family participate in coordinating the synergistic expression of key components in the MHC class I and class II pathways [67]. The positive selection sites of these two genes also demonstrate that the NLR gene family plays an important role in coordinating MHC molecule immune processes. Research indicates that *NLRP3* enhances tolerance by modulating MHC class I to attenuate negative immune responses [157,158]. Positive selection sites for bat *NLRP3* within the MHC functional domain may indicate that it attenuates adverse reactions to viral resistance by inhibiting MHC signaling. Its specific functions and involvement in immune processes require further investigation. molecular evolutionary patterns.

## 5. Conclusions

This study is the first to analyze the molecular evolution of the NLR gene family in Chiroptera species. A total of 572 NLR genes were identified in 26 species, including 501 complete genes and 71 pseudogenes. The results of the selection pressure analysis showed that, except for the *NLRP1* gene in Vespertilionidae, NLR genes in Chiroptera were subject to purifying selection pressure overall. This indicates that the NLR gene family has high stability and functionality. The *NLRP1* gene has been subject to positive selection pressure in Vespertilionidae, and there are also many sites of positive selection. This may be related to the complex feeding habits of bat species and their exposure to a wide variety of viruses. The NLR gene family is highly conserved during evolution, and this high degree of conservation ensures functional stability to better resist viruses. There are a large number of positive selection sites in the NLR genes of bats, which is closely related to the strong viral pressure they are experiencing. The number of positive selection sites is highest in Rhinolophidae and Hipposideridae, and the *NLRX1* may play an important role in the antiviral processes of these two species. The evolutionary characteristics of multiple genes indicate that the NLR gene family in bats primarily enhances innate immune function by regulating the type I IFN signaling pathway and the NF-κB signaling pathway. Positive selection sites for some genes are in immune-related domains. Positive selection sites for *NLRP1* and *NLRP3* indicate that the NLR gene family plays an important role in coordinating the immune process of MHC molecules. Based on this study and recent related research, we found that Hipposideridae may have stronger viral load capacity. In subsequent studies on virus transmission, more attention should be paid to the role of Hipposideridae in the virus transmission process. This study also found that the evolutionary rate of bat virus-related resistance genes and the prevalence of positive selection sites vary depending on different environments, viral pressures, and dietary differences. This also means that bats have different specific strategies for resisting viruses, demonstrating the adaptability and diversity of bats’ antiviral capabilities. The NLR gene family enhances the innate immune capacity of bats through a combination of adaptive evolution of some genes to enhance gene functionality and maintenance of gene conservatism at a low evolutionary rate. The specific role played by the relevant genes in the antiviral capacity of bats still needs to be further verified in experiments.

## Data Availability

The data provided in this study can be found in the NCBI Genome Database. Specific genomic information is provided in Supplementary Table S1. The gene screening methods have been explained in our article. Further inquiries can be directed to the corresponding author.

## Supplementary Materials

The following supporting information can be downloaded at https://www.mdpi.com/article/10.3390/biom15121715/s1. Table S1: Genomic information for Chiroptera species; Table S2: Analysis of positive selection sites for Chiroptera species; Table S3: Analysis results of the Chiroptera species Branch Model; Table S4: Positive selection for NLR gene branch locus models in Chiroptera; Table S5: The location of positively selected sites within functional domains and their corresponding functional implications.
