# Supplementary material for: Molecular Evolution of the NLR Gene Family Reveals Diverse Innate Immune Strategies in Bats
Source: Biomolecules. 2025 Dec 10;15(12):1715. doi: 10.3390/biom15121715 (PMC12730308; doi:10.3390/biom15121715)
Supplement: Supplementary file 1 [file biomolecules-15-01715-s001.zip › Table S1.pdf]

Table S1.Genomic information for Chiroptera species

| Species | Genome Assembly Number | Depth (×) |
|---|---|---|
| ***Murina aurata*** | GCA_004026665.1 | 33.3 |
| ***Pipistrellus nathusii*** | GCA_963693515.1 | 42.0 |
| ***Myotis planiceps*** | GCA_036250175.1 | 30.0 |
| ***Myotis vivesi*** | GCA_035771395.1 | 30.0 |
| ***Pipistrellus pygmaeus*** | GCA_949987585.2 | 71.0 |
| ***la io*** | GCA_025583905.1 | 260.0 |
| ***Eptesicus nilssonii*** | GCA_030846915.1 | 141.0 |
| ***Nyctalus aviator*** | GCA_036971965.2 | 380.0 |
| ***Trachops cirrhosus*** | GCA_028533065.1 | 106.0 |
| ***Phyllostomus hastatus*** | GCA_019186645.2 | 60.0 |
| ***Sturnira hondurensis*** | GCA_014824575.2 | 158.0 |
| ***Rhynchonycteris naso*** | GCA_037038555.1 | 56.7 |
| ***Saccopteryx bilineata*** | GCA_036850765.1 | 70.0 |
| ***Pteropus alecto*** | GCA_000325575.1 | 110.0 |
| ***Eidolon helvum*** | GCA_000465285.1 | 18.0 |
| ***Aselliscus stoliczkanus*** | GCA_043727835.1 | 32.0 |
| ***Hipposideros larvatus*** | GCA_031876335.1 | 30.47 |
| ***Hipposideros pendleburyi*** | GCA_021464545.1 | 50.25 |
| ***Hipposideros armiger*** | GCA_001890085.1 | 218.6 |
| ***Rhinolophus ferrumequinum*** | GCA_004115265.3 | 52.8 |
| ***Rhinolophus affinis*** | GCA_005887515.2 | 239.0 |

| | | |
|---|---|---|
| ***Rhinolophus sinicus*** | GCA_036562045.1 | 87.1 |
| ***Rhinolophus rex*** | GCA_041825415.1 | 50.0 |
| ***Rhinolophus trifoliatus*** | GCA_043728145.1 | 27.0 |
| ***Rhinolophus luctus*** | GCA_000003025.6 | 65.0 |
| ***Rhinolophus hipposideros*** | GCA_964194215.2 | 39.0 |
