# Supplementary material for: Molecular Evolution of the NLR Gene Family Reveals Diverse Innate Immune Strategies in Bats
Source: Biomolecules. 2025 Dec 10;15(12):1715. doi: 10.3390/biom15121715 (PMC12730308; doi:10.3390/biom15121715)
Supplement: Supplementary file 1 [file biomolecules-15-01715-s001.zip › Table S2.pdf]

Table S2. Analysis of positive selection sites for Chiroptera species

Family	Gene	InL M7	InL M8	df	p-Values	BEB	SLAC	FEL	REL
Vespertilionidae	CIITA	-3477.59	-3478.83	2	0.2893		63 253	8 59 120 315 444 518 548	127 188 250 433
	NAIP	-4076.463	-4064.53	2	6.58E-06**	39 H 0.984**<br>250 G 0.974*<br>285 T 0.968*<br>432 H 0.993**<br>433 I 0.995**	42 44 ***250*** 556	***39*** 41 45 222 ***250*** ***285*** 429 430 ***432*** ***433*** 437 528 564 570	40 128 217 ***433*** 507 517 555
	NOD1	-3970.42	-3972.40	2	0.135		334 352	243 291 301 430	116 197 361 362
	NOD2	-2882.236	-2883.236	2	0.367		7 101	34 48 73 147 165 166 173	107 123 182 199 201 202 223 258
	NLRC3	-2113.41	-2113.42	2	0.994		142	154 212 227 236	148 153 211
	NLRC4	-4739.53	-4739.05	2	0.623			128 330 384 469	443 459 472 491 517 632
	NLRC5	-5717.19	-5717.05	2	0.869		161	201 295 419	61 160
	NLRP1	-5928.13	-5879.18	2	5.51E-22**	8 G 0.996**<br>10 G 0.972*<br>12 K 0.995**<br>13 A 0.972*<br>14 L 0.968*<br>18 L 0.961*<br>19 R 0.965*<br>23 D 0.989*<br>24 L 0.985*<br>25 I 0.990**	3 ***8*** ***19*** ***72*** ***217***	1 2 4 9 ***10*** ***13*** ***14*** 15 16 17 ***18*** ***19*** 20 21 22 ***23*** ***24*** ***25*** ***26*** ***27*** ***28*** 29 ***30*** 31 32 ***33*** ***34*** ***35*** ***36*** ***62*** ***63*** 64 65 66 ***67*** 70 71 ***72*** ***74*** ***75*** ***76*** ***77*** 78 ***79*** ***80*** 82 83 84 85 87 88 89 ***102*** 103 104 ***106*** 109 111 115	5 6 7 ***8*** ***10*** 11 ***12*** ***13*** ***14*** ***34*** ***35*** ***36*** 39 40 42 43 44 46 ***47*** 48 49 ***50*** 51 52 ***53*** ***54*** 55 56 ***57*** ***58*** 59 ***60*** 61 ***62*** ***63*** 93 94 95 96 99 101 ***102*** 153 158 173 ***217*** 225 228

26 Q 0.979*<br>27 L 0.987*<br>30 S 0.972*<br>33 R 0.970*<br>34 D 0.993**<br>35 A 0.963*<br>36 G 0.963*<br>47 S 0.991**<br>50 G 0.971*<br>53 R 0.980*<br>54 L 0.974*<br>57 V 0.958*<br>58 G 0.953*<br>60 G 0.982*<br>62 T 0.978*<br>63 S 0.996**<br>67 Q 0.951*<br>72 M 0.957*<br>74 S 0.993**<br>75 N 0.951*<br>76 N 0.999**<br>77 I 0.993**<br>79 S 0.993**<br>80 E 0.969*<br>102 N 0.993**<br>106 N 0.976*	***117*** 118 119 122 125 140 177 178 179 180 181 182 185 188 189 ***191*** ***193*** ***194*** ***217*** 255 256 294 299

					117 G 0.998**<br>191 R 0.978*<br>193 S 0.955*<br>194 N 0.970*<br>217 S 0.971*			
NLRP2	-2273.55	-2268.42	2	0.005**			6 30 75 104 129 130 200	197 201 205
NLRP3	-3290.57	-3290.58	2	0.980		71 489	9 59 145	417
NLRP4	-2980.94	-2971.47	2	7.71E-05**		17 19	22	5 57 76
NLRP5	-8265.34	-8260.18	2	0.006**		209 336	169 171 256 262 290 312 319 320 322 326	95 105 118 138 330
NLRP6	-2030.57	-2030.18	2	0.677			213	158 198 259
NLRP7	-2660.65	-2660.67	2	0.898		76	78 123 141 176	128 153 156 158 159 161 162
NLRP8	-5527.18	-5524.13	2	0.047*		145 213	176 179 216 219 250 258 288 279 306	55 85 89 339
NLRP9	-2165.73	-2163.19	2	0.079		48 219	24 56 57 58 96 256 257 258 260	151 198 199 200 201
NLRP10	-4381.23	-4377.91	2	0.036*		53 101 242	162 164 300 529	4 5 6 90 501 507 509 511
NLRP11	-5406.51	-5403.66	2	0.057			3 6 8 36 79	49 58
NLRP12	-1319.96	-1320.90	2	0.368		8 145 342	26 83 96 216 217	71 72 75 76 323 325 326 328 330
NLRP13	-2510.28	-2510.04	2	0.787		35 36	59 98 118 226	198

	NLRP14	-1970.69	-1970.63	2	0.941		170 189 190	218 219 353 356	58 69 70 76 114 154 232 256 257 258 291 300
	NLRX1	-6508.18	-6497.34	2	1.96E-05**		48 52 145 217	1 6 8 24 159 190 208 209 213 214 218 220 223 224 225 226 227 231232 235 244 254 331	37 47 53 57 60 63 65 67 85 86 360 446
	CIITA	-2524.09	-2521.05	2	0.047*		70 165	120 123 137 181 462	16 63
	NAIP	-2904.76	-2904.45	2	0.733		150 569	189 190 198 199 422 436 438 439 489 490 500 501 503 504 505	211 242 256 257 258 299 301 309
	NOD1	-2600.67	-2600.11	2	0.571			63 284	103
Emballonuridae	NOD2	-3603.19	-3603.28	2	0.914		136 138 139	156 158 166 170 236 238 256 299	2 5 6 123 128 301 333 366 389 421 427
	NLRC3	-1023.79	-1023.64	2	0.860		256 278	142 333 335 336 338 345 369	298 301 306
	NLRC4	-3869.24	-3781.08	2	5.16E-39**	1 I 0.999** 3 E 0.995** 6 Q 0.999** 8 L 0.994** 9 I 0.995** 10 Q 0.995**	***3*** ***41*** ***82***	37 ***38*** 39 40 ***41*** ***42*** ***44*** ***45*** 46 47 ***51*** ***52*** ***53*** 54 55 152 186 303 312 500 516 538 570	***1*** 2 ***3*** 4 5 ***6*** ***8*** ***9*** ***10*** ***12*** ***18*** ***19*** 20 21 22 23 25 ***26*** 27 28 ***31*** 32 33 ***34*** 35 36 37 ***38*** 39 40 ***41*** ***42*** ***44*** ***45*** 46 47 48 50 ***51*** ***52***

					12 M 0.973*<br>18 K 0.994**<br>19 Q 0.988*<br>26 E 0.998**<br>31 N 0.993**<br>34 E 0.995**<br>38 I 0.977*<br>41 E 0.997**<br>42 K 0.981*<br>44 E 0.995**<br>45 Q 0.993**<br>51 V 0.992**<br>52 I 0.977*<br>53 H 0.996**<br>60 S 0.997**<br>77 F 0.996**<br>80 L 0.992**<br>81 N 0.991**<br>82 G 0.999**			***53*** 54 55 57 58 59 ***60*** 61 74 75 76 ***77*** 78 79 ***80*** 81 ***82*** 83 84 624 634 657 684 703 729
NLRC5	-3568.33	-3554.58	2	1.07E-06**	1 A 0.988*<br>5 C 0.986*	564 663	***1*** 3 ***5*** 7 9 453 491	18 25 70 134 175 214 650
NLRP2	-2608.23	-2606.19	2	0.130			248 293	93 116 169 188
NLRP3	-4997.01	-4983.80	2	1.84E-06**	728 F 0.979*<br>736 N 0.953*<br>763 G 0.989*	***728*** 733 ***736***	210 247 738 739 743 750 752 753 758 759 ***763*** 847	718 721 722 725 726 ***728***
NLRP4	-2483.18	-2483.26	2	0.923		119 300	189 216 217 326	401 403 443

NLRP5	-3035.49	-2998.20	2	2.30E-224**	3 W 0.991**<br>11 K 0.998**<br>12 K 0.980*<br>14 E 0.976*<br>16 E 0.995**<br>17 D 0.989*<br>18 I 1.000**<br>19 D 0.999**<br>24 I 0.997**<br>25 A 0.993**<br>26 A 0.998**<br>28 G 0.997**<br>29 G 0.997**<br>32 G 0.999**<br>33 Y 0.997**<br>36 I 0.991**<br>131 N 0.976*<br>384 H 0.988*	***18*** ***19*** ***26*** ***27***	1 ***3*** 5 6 ***11*** ***12*** 13 ***14*** ***16*** ***17*** ***18*** ***19*** 30 31 ***32*** ***33*** ***36*** 37 68 ***131*** 184 361 ***384***	21 22 ***24*** ***25*** ***26*** 27 ***28*** ***29*** 30 31 ***32*** ***33*** 34
NLRP6	-2671.96	-2671.42	2	0.582		359	288 320 338 346 472	121 154 527
NLRP8	-3961.91	-3959.08	2	0.059		127 128 342 437	103 160 276 282 292 296 297 303 304 308 321 331 338 389 390 391 398 412 419 426 439 440 462 465	201 220 231 240 245 257 266 267 273 368 342 368 380 427 432 436 459

								484 489 491 515 519 525 529 531 553	
	NLRP9	-4057.93	-4056.70	2	0.293		23 36	2 4 9 52 54	43 64 72 74 123 130
	NLRP10	-2618.27	-2617.48	2	0.453			135 157	49 65 117 131
	NLRP12	-4852.74	-4852.75	2	0.990		73 76 115	269 280 299 304 532 567 598 601	355 369 446 447 498 500 632 656 668 670 682
	NLRP13	-4687.94	-4653.79	2	1.48E-15**	102 E 0.995**<br>106 P 0.988*<br>107 S 0.988*<br>108 C 0.994**<br>109 S 0.996**<br>114 F 0.968*<br>115 S 0.996**	103 104 ***106*** ***107***	***106*** ***107*** ***108*** ***109*** 110 ***114*** ***115*** 116 357 550 568 887	1 9 63 99 100 101 ***102*** 103 110 111 ***114*** ***115*** 915
	NLRP14	-5104.64	-5104.65	2	0.990		168 157 646	520 581 670 698 1030	458 461 832 856 923 965 996 1020
	NLRX1	-4704.32	-4704.13	2	0.825		426	36 72 73 165 169 432 444 763 765 766	819 844
	CIITA	-2461.46	-2458.48	2	0.051		104 165	82 99 253 256	244 383 481
Pteropodidae	NAIP	-6592.22	-6360.71	2	2.9E-101**	3 Q 0.998**<br>4 E 0.973*<br>6 A 0.996**<br>10 K 0.961*<br>18 T 0.979*	***18*** ***22*** ***101*** ***925*** ***928***	***31*** ***32*** ***39*** ***42*** ***49*** ***56*** ***61*** ***67*** ***80*** ***183*** ***184*** 185 187 ***194*** ***195*** ***199*** ***214*** ***217*** ***222*** ***233*** ***238*** 400 403	***3*** ***4*** ***6*** ***10*** 15 16 17 18 50 ***61*** ***67*** ***80*** ***214*** ***217*** ***222*** ***233*** ***238*** ***244*** ***246*** ***253*** ***258*** ***260*** ***262*** ***269*** ***270*** ***272***

22 L 0.967*<br>31 V 0.985*<br>32 Q 0.958*<br>39 E 0.974*<br>42 Q 0.975*<br>49 K 0.975*<br>56 R 0.973*<br>61 R 1.000**<br>67 T 0.981*<br>80 A 0.956*<br>101 R 0.995**<br>183 F 0.967*<br>184 A 0.968*<br>194 F 0.970*<br>195 P 0.964*<br>199 P 0.968*<br>214 K 0.975*<br>217 I 0.962*<br>222 P 0.970*<br>233 Y 0.971*<br>238 A 0.965*<br>244 E 0.956*<br>246 W 0.976*<br>253 V 0.968*<br>258 L 0.996**<br>260 A 0.967*	459 512 622 ***726***<br>***740*** 743 745 761<br>773 ***779*** ***878*** ***881***<br>***933*** ***963***	***297*** ***306*** ***312*** ***313***<br>372 388<br>***575*** ***577*** ***726*** ***740***<br>***779*** 801 803 806<br>818 836 ***878*** ***881***

					262 F 0.961*<br>269 V 0.989*<br>270 Q 0.976*<br>272 F 0.951*<br>297 N 0.962*<br>306 A 0.961*<br>312 A 0.979*<br>313 D 0.968*<br>575 R 0.963*<br>577 Q 0.967*<br>726 R 0.966*<br>740 S 0.960*<br>779 C 0.968*<br>878 E 0.998**<br>881 F 0.963*<br>925 Q 0.977*<br>928 K 0.975*<br>933 L 0.963*			
NOD1	-2502.92	-2501.82	2	0.332		432 436	75 93 94 181 186 187 188 356 389 446 462	223 269 270 281 288 581
NOD2	-2709.99	-2709.97	2	0.980			224 263 284 285 318 333 335 369	118 278 341
NLRC4	-3838.93	-3783.69	2	1.02E-24**	9 R 0.987*<br>11 V 0.954*<br>12 N 0.999**	***16*** ***18*** ***19*** ***53***	***9*** ***11*** ***12*** ***13*** ***14*** ***16*** ***18*** 20 21 22 ***23*** ***29*** ***32*** ***35*** ***36*** 38 39 ***43*** ***44***	2 3 ***9*** ***11*** ***12*** ***13*** ***14*** ***67*** ***68*** ***70*** ***72*** 222 225 282 336 349 353

					13 S 0.998**<br>14 R 0.987*<br>16 I 0.956*<br>18 T 0.999**<br>19 G 0.998**<br>23 T 0.979*<br>29 Y 0.994**<br>32 V 0.952*<br>35 L 0.993**<br>36 V 0.969*<br>43 L 0.993**<br>44 A 0.995**<br>46 L 0.973*<br>47 K 0.988*<br>53 R 0.980*<br>67 I 0.975*<br>68 V 0.970*<br>70 S 0.996**<br>72 T 0.979*		***46*** ***47*** 123 40 145<br>173 718 721	365 373 389 395<br>445 447 615 616<br>700 713 715 733
NLRC5	-2700.78	-2697.70	2	0.045*		54	149 176 223 321<br>332 357 374 437449<br>452	99 247 254 283
NLRP3	-2608.98	-2608.88	2	0.904		281 289 332	89 96 132 246 279	177 186
NLRP5	-2440.78	-2440.57	2	0.811		244 245	84 85 116 119 126<br>288 489 494 504	3 6 25 45 107 160<br>175 238 244 317<br>327 337 450 524530

									532
	NLRP6	-2559.10	-2556.95	2	0.116		467 584	132 148 184 316 332	446 458 459
	NLRP10	-2569.67	-2565.12	2	0.011*			1 93	277 334 395
	NLRP14	-2376.01	-2375.08	2	2.92E-83**		97 99 282 293	4 8 27 254 255 428 482 485	77 268 274 383 387 403 408
	NLRX1	-2497.90	-2490.65	2	0.0007**	2 S 0.965* 538 V 0.955*	1 ***2*** ***538*** 539	***538*** 19 23 63 193 200 243 317 446 540 541 542	***2*** 496 501 ***538***
Phyllostomatidae	CIITA	-5585.65	-5543.77	2	6.48E-19**	190 T 0.975* 1024 P 0.959* 1025 A 0.981* 1027 S 0.955* 1028 L 0.992** 1034 H 0.961* 1035 G 0.973* 1036 E 0.955* 1045 V 0.995** 1046 C 0.990* 1047 P 0.997** 1048 L 0.973* 1054 F 0.993** 1055 P 0.961*	***190*** 577 651	92 175 189 ***190*** 200 709 710 730 757 786 962 965 ***1024*** ***1025*** ***1027*** ***1028*** ***1034*** ***1035*** ***1036*** 1042 1043 1044 ***1045*** ***1046*** ***1047*** ***1048*** 1050 1501 ***1054*** ***1055*** 1056	272 294 306 355 834 ***1024*** ***1025*** ***1027*** ***1028*** ***1034*** ***1035*** ***1036*** 1037 1038 1039 1040 1041
	NAIP	-3114.24	-3114.28	2	0.960		281	79 80 81 137 138 164 166 167 168	77 262 361 362
	NOD1	-4491.69	-4488.47	2	0.039*		226 436 439	327 436 469 507	46 201534 536 537

							508	574 576 639 640
NOD2	-3117.13	-3117.14	2	0.990			173 218 588	437
NLRC3	-2227.28	-2217.70	2	6.91E-05**	285 P 0.986*<br>287 E 0.971*<br>293 K 0.978*<br>294 E 0.952*<br>296 I 0.987*<br>297 K 0.995**	***285*** ***287*** ***293*** ***294***	132 280 281 283 ***285*** 286 ***287***	5 65 290 292 ***293*** ***294*** 295 ***296*** ***297*** 298 373 389
NLRC4	-5241.36	-5241.37	2	0.990			109 227 467 635	889
NLRC5	-3039.73	-3036.46	2	0.037*		257 344	263 317 364 401 402	77 183 321 336
NLRP1	-4408.20	-4405.17	2	0.048*	276 L 0.955*	139 383 457	48 54 184	***276*** 277 287 422 434 436 438
NLRP3	-3042.05	-3036.99	2	0.006**	7 K 0.953*	6 ***7***	***7*** 92 579	***7*** 8
NLRP4	-4021.55	-4018.38	2	0.042*		142 187 321 482	34 37 41 83 94 103 125 127 231 244 251 268 292 293 367 383 384 394 396 412 413 459 469 471 472 522	45 50 54 53 72 205 208 213 221 224 429 437 441449 456 486 489 493 494 517
NLRP5	-8062.94	-8054.36	2	0.0002**	1 Q 0.958*<br>16 V 0.951*<br>47 T 0.956*<br>199 R 0.962*<br>1023 A 0.984*	***47*** ***199***	***1*** 3 7 11 ***16*** 509 671 685 694 712 723 ***1023***	***1*** ***16*** 21 25 35 17 752 774 780 827 1017 1018 1021 1022 ***1023***
NLRP6	-3082.56	-3080.08	2	0.083		356 412	72 171 177	441 456 462 482

								494 500 509
NLRP7	-5826.93	-5785.05	2	6.48E-19**	107 V 0.959*<br>286 A 0.967*<br>420 R 0.971*<br>520 D 0.975*<br>523 H 0.959*<br>527 D 0.957*<br>528 L 0.984*<br>529 C 0.989*<br>530 S 0.995**<br>533 S 0.967*<br>535 N 0.963*<br>537 S 0.960*<br>539 S 0.992**<br>873 A 0.984*<br>882 S 0.974*<br>889 Q 0.988*<br>895 N 0.958*<br>904 E 0.980*<br>908 L 0.988*<br>914 R 0.993**<br>919 F 0.992**<br>921 R 0.977*<br>922 R 0.967*<br>928 N 0.995**<br>930 L 0.984*	5 53 ***286*** ***895*** ***934***	175 235 256 276 279 282 ***286*** ***420*** 433 467 471 510 516 518 519 ***520*** ***523*** ***527*** ***528*** ***529*** ***530*** 531 ***533*** ***535*** ***537*** ***539*** 764 785 832 843 851 870 871 ***873*** 876 877 879 ***882*** 883 884 886 ***889*** 897 898 ***904*** ***908*** ***914*** ***919*** ***928*** ***930*** ***934*** 936 946 ***952*** ***955*** 956 959 961 ***964*** ***973*** ***974*** 976 ***979*** 981	93 100 105 ***107*** 111 306 370 396 ***420*** ***520*** ***523*** ***527*** ***528*** ***529*** ***530*** 726 727 ***889*** 890 892 893 ***895*** ***914*** ***919*** ***921*** ***922*** 924 926 ***928*** ***930*** *971* ***973*** ***974***

					934 Y 0.994**<br>952 C 0.951*<br>955 K 0.952*<br>964 K 0.961*<br>973 L 0.982*<br>974 D 0.978*<br>979 T 0.963*			
NLRP8	-4155.19	-4155.14	2	0.946		156 198 199 201	46 89 133 134 166<br>178	256 287 288 306<br>309 404 405 406<br>408 40 9410 456
NLRP9	-3807.24	-3793.88	2	1.58E-06**	6 D 0.952*<br>36 P 0.957*<br>41 K 0.955*<br>46 E 0.959*<br>49 A 0.979*<br>58 G 0.962*<br>73 N 0.983*	***6*** ***41*** ***46*** ***58*** ***73***	***6*** 12 14 15 16 22 23<br>24 26 27 29 30 31<br>34 ***36*** ***49*** 50 53 54<br>56 57 ***58*** ***73*** 103 105<br>121 123 195 314<br>315 338 365 446<br>448 515 523 538<br>545	1 2 3 4 5 ***6*** 7 8 ***36*** 37<br>***41*** 43 44 45 ***46*** 48<br>***49*** ***73*** 408 461 484<br>488
NLRP10	-2744.91	-2731.96	2	2.38E-06**	6 L 0.955*<br>7 N 0.985*<br>498 N 0.973*	***7*** ***493*** ***498***	1 2 4 ***6*** 317 328	***6*** ***7*** 307 493
NLRP11	-2786.12	-2785.58	2	0.582			151 172 177	170 181
NLRP12	-2843.28	-2843.32	2	0.961		776 786	58 323 356 389<br>1003 1048 1192	218 219 236 239<br>987 988 993
NLRP13	-3044.73	-3044.92	2	0.827			37 176	147 446 475
NLRP14	-2802.63	-2802.14	2	0.612			154 398 475 491	19 31

Rhinolophidae	NLRX1	-4324.87	-4322.95	2	0.146		40 45 257 508	8 22 157 773 826 893	170 409 410 482 484 496 756
	CIITA	-7215.64	-7214.13	2	0.2212		45 86 278 572	60 62 69 74 79 90 91 119 120 122 123 124 126 127 129 131 132 310 376 377 501 572	7 8 16 20 102 104 106 109 203 226 572 619 629
	NAIP	-1225.01	-1224.89	2	0.8962		120 128	225 229 231 365 368	48 128
	NOD1	-1115.34	-1112.605	2	0.021*2	514 V 0.953*	234 ***514***	46 283 284	288 292 330 ***514*** 516
	NOD2	-4770.10	-4732.73	2	5.89E-172**	1 L 0.958* 2 F 0.995** 4 C 1.000** 5 L 0.993* 6 L 1.000** 7 D 0.999** 230 K 0.976*	***1 2 4 5***	***4 5 6 7 230*** 324 342	***1 2*** 3 ***4 5 6 7***
	NLRC3	-1115.34	-1112.605	2	0.0652		168 196	41 85 123 125 268 266 289 342 356 432	81
	NLRC4	-4858.62	-4862.79	2	0.015*2	7 Q 0.964* 11 Q 0.966* 29 E 0.954* 61 F 0.958* 64 S 0.966*		***7 11 29 79*** 77 78 79 80 88 ***147*** 370 376	***61 64 68***

					68 W 0.972*<br>79 Q 0.966*<br>147 S 0.960*			
NLRC5	-4210.01	-4201.59	2	0.00022	1 A 0.998**<br>201 R 0.971*<br>249 H 0.988*	***201***	***1*** 18 21 151 158 178 184 ***201*** 317 357 367 424 454 464 513	***201*** ***249***
NLRP1	-4901.26		2	8.40E-121**	4 K 0.961*<br>9 S 0.965*<br>11 D 0.953*<br>22 V 0.974*<br>25 Q 0.968*<br>33 L 0.988*<br>165 E 0.969*<br>208 R 0.994**<br>393 I 0.962*<br>435 G 0.994**<br>440 W 0.995**<br>447 Q 0.972*<br>477 C 0.984*<br>503 M 0.998**	***33*** ***393*** 394 397	***4*** ***9*** 10 ***11*** 12 14 15 19 ***22*** ***25*** 31 ***33*** ***447*** ***477*** ***503*** 505 510 521	162 ***165*** ***208*** 240 241 245 304 ***393*** 419 ***435*** 436 ***440*** 444 ***447*** ***477*** 480 482 484 ***503***
NLRP2	-2265.08	-2265.07	2	0.9920			22 197 202	272
NLRP3	-4234.62	-4232.85	2	0.1722		37 389	231 266 323 325 377	156 158 201
NLRP4	-5348.28	-5348.27	2	0.9929		413	76 96 99 102 105 232 256 289 312	144 145 165 188

							356 388 400	
NLRP5	-5114.67	-5114.66	2	0.9920		102 316	243 244 290 291	86 89 90 89 101 256 257 289 301 303 342
NLRP6	-8766.33	-8744.55	2	3.51E-210**		384 408 440 518	44 149 150 323 327 330 332 364 373	456 457 458 459
NLRP7	-3671.43	-3669.84	2	0.2204		76 355 432	18 22 39 40 396 399	87 119 192 263 267 290 293 295 320 342 448 450 456 487 518
NLRP8	-3240.75	-3240.25	2	0.607		35	345 444	298
NLRP9	-3907.47	-3906.95	2	0.594		94 346 522	217 225 234 271 294 299 501 505 507 508 511 512 513 514 517 518 519	24 353 449 456 473 474 476 481 482 485 490 493 495
NLRP10	-4344.77	-4337.23	2	0.0005**	482 M 0.980* 501 K 0.991** 511 C 0.993** 513 T 0.972* 514 G 0.982* 517 G 0.962* 522 T 0.961*	331 ***482*** ***517*** ***522***	16 24 46 56 89 159 234 271 346 353 385 392 ***501*** ***511*** 512 ***513*** ***514*** ***517*** ***522***	94 201 206 213 214 217 221 225 292 294 299 ***501*** ***511*** ***513*** ***514*** 515 ***517*** 518 519 520 ***522***
NLRP11	-11470.38	-11142.79	2	3.51E-143**		763 1011 1056 1059	238 269 1138 1249 1589 1632	158 473 561 562 566
NLRP12	-3678.77	-3676.99	2	0.169			162 222 411 519	56 61

							537 543 555	
NLRP13	-3600.84	3586.84	2	8.32E-07**	1 G 0.989*<br>2 E 0.997**<br>3 F 0.997**<br>192 S 0.976*<br>452 M 0.995**	***192*** ***452***	***1*** ***2*** ***3*** 48 268 401 421 447 448 196	***1*** ***2*** ***3*** 377 399
NLRP14	-3720.13	-3720.14	2	0.990			383	11 432 433 456 780
NLRX1	-3113.19	-3096.01	2	3.46E-08**	6 I 0.987*<br>8 A 0.958*<br>9 L 0.984*<br>10 K 0.975*<br>14 P 0.984*<br>15 L 0.976*<br>16 Q 0.993**<br>19 G 0.954*<br>24 S 0.961*<br>25 G 0.953*<br>27 P 0.969*<br>32 E 0.971*<br>33 Q 0.962*<br>36 R 0.964*<br>42 E 0.990**<br>48 E 0.970*<br>51 Q 0.959*<br>52 F 0.956*<br>56 L 0.966*	***32*** ***33*** ***87*** ***92*** ***107*** 154 ***155*** ***156***	2 3 4 ***6*** ***8*** ***9*** ***10*** 12 13 ***14*** ***15*** ***16*** 28 30 31 35 ***36*** ***42*** ***92*** ***94*** ***95*** 97 98 ***99*** 100 101 103 ***104*** ***105*** 106 ***107*** 171 186 207 218 230 240	***14*** ***15*** ***16*** 17 ***19*** 20 23 ***24*** ***25*** 26 ***27*** ***36*** 37 38 39 40 41 ***42*** 45 ***48*** 49 ***51*** ***52*** 53 54 ***56***57 58 ***59*** 61 62 ***63*** 64 65 66 ***67*** ***68*** 69 70 71 72 ***73*** 74 ***77*** ***78*** ***80*** ***107*** 108 109 111 ***112*** 114

						59 V 0.985*<br>63 P 0.956*<br>67 E 0.950*<br>68 S 0.963*<br>73 L 0.964*<br>77 S 0.980*<br>78 E 0.962*<br>80 A 0.986*<br>87 T 0.997**<br>92 A 0.992**<br>94 S 0.965*<br>95 Q 0.973*<br>99 P 0.967*<br>104 R 0.980*<br>105 R 0.954*<br>107 Q 0.951*<br>112 Y 0.979*<br>155 A 0.975*<br>156 S 0.988*			
Hipposideridae	CIITA	-5586.72	-5576.23	2	2.76E-05**	1 P 0.982*<br>2 V 0.973*<br>12 L 0.988*<br>25 S 0.963*<br>37 N 0.969*<br>51 N 0.992**<br>75 P 0.955*	***12*** ***146*** ***163*** ***171***	***1*** ***2*** ***25*** 32 ***37*** ***51*** ***75*** ***146*** ***163*** ***171***	***51*** ***75*** 136 155 ***163*** ***171***

NAIP	-7688.76	-7648.77	2	4.29E-18**	146 P 0.951*<br>163 H 0.953*<br>171 I 0.980*<br>8 T 0.953*<br>12 S 0.988*<br>16 L 0.993**<br>18 C 0.964*<br>19 F 0.979*<br>24 C 0.960*<br>25 S 0.957*<br>29 P 0.977*<br>35 V 0.980*<br>38 C 0.978*<br>41 C 0.995**<br>43 N 0.976*<br>45 Y 0.978*<br>56 S 0.969*<br>57 F 0.952*<br>59 S 0.984*<br>61 V 0.965*<br>62 S 0.974*<br>65 R 0.958*<br>76 S 0.953*<br>79 W 0.959*<br>81 F 0.968*<br>87 F 0.954*	***19*** ***332*** ***335*** ***769*** ***927***	***18*** ***19*** 21 22 ***24*** ***25***<br>27 28 ***29*** ***35*** ***38*** ***41***<br>***43*** ***45*** ***57*** ***59*** ***102*** ***104***<br>***105*** 107 ***120*** ***121***<br>***122*** ***128*** ***132*** ***149***<br>***153*** ***154*** ***164*** ***170***<br>***172*** ***173*** ***175*** ***194***<br>***198*** ***202*** ***207*** ***211***<br>***214*** ***217*** ***220*** ***222***<br>***223*** ***224*** ***225*** ***226***<br>***230*** ***233*** ***236*** ***242***<br>***244*** ***245*** ***247*** ***249***<br>***251*** ***252*** ***254*** ***257***<br>***260*** 261 263 268<br>270 ***288*** ***295*** ***296***<br>***301*** ***307*** ***310*** ***332***<br>***335*** ***342*** ***344*** ***347***<br>***350*** ***769*** ***773*** 920<br>***925*** 927	***8*** 11 ***12*** 13 14 15 ***16***<br>17 ***18*** ***19*** ***61*** ***62*** ***65***<br>***76*** ***79*** ***81*** ***87*** ***88*** ***97***<br>***98*** ***102*** ***104*** ***105*** 180<br>185 186 188 189<br>190 ***202*** ***207*** ***211***<br>***214*** ***217*** ***276*** ***277***<br>278 280 84 285 287<br>***288*** ***295*** ***296*** ***301***<br>***307*** ***310*** 311 312<br>315 326 328 ***332***<br>***335*** ***342*** ***344*** ***347***<br>***350*** 599 717 753<br>766 800 842 853<br>857 886 896 899

88 F 0.967*

97 S 0.977*

98 H 0.967*

102 P 0.993**

104 Q 0.979*

105 G 0.988*

120 P 0.966*

121 T 0.965*

122 Q 0.967*

128 K 0.955*

132 L 0.963*

149 Q 0.986*

153 P 0.989*

154 I 0.958*

164 I 0.984*

170 V 0.955*

172 V 0.960*

173 Y 0.966*

175 F 0.968*

194 I 0.959*

198 Q 0.976*

202 A 0.957*

207 Q 0.999**

211 T 0.998**

214 L 0.972*

217 P 0.997**

220 T 0.974*

222 I 0.994**

223 N 0.979*

224 G 0.996**

225 E 0.951*

226 L 0.999**

230 Q 0.993**

233 I 0.988*

236 R 0.993**

242 G 0.958*

244 W 0.995**

245 A 0.952*

247 G 0.990**

249 V 0.951*

251 T 0.970*

252 S 0.972*

254 Q 0.989*

257 V 0.970*

260 P 0.970*

276 S 0.973*

277 P 0.966*

288 E 0.960*

295 K 0.975*

296 S 0.964*

301 L 0.961*

307 S 0.971*

					310 S 0.971*<br>332 F 0.968*<br>335 F 0.968*<br>342 S 0.993**<br>344 V 0.954*<br>347 Y 0.985*<br>350 P 0.951*<br>769 F 0.982*<br>773 W 0.956*<br>925 C 0.986*<br>927 A 0.966*			
NOD1	-3393.04	-3386.55	2	0.0015*		120 247 330	252 283 288 332<br>456 514	292 516
NOD2	-7660.78	-7368.03	2	7.21E-128**	1 S 1.000**<br>3 L 0.996**<br>4 P 1.000**<br>5 V 0.975*<br>7 V 0.999**<br>12 S 0.994**<br>13 K 0.998**<br>14 G 0.990**<br>17 L 0.990**<br>18 G 0.999**<br>20 A 0.999**<br>22 T 0.964*<br>23 P 0.998**	***196*** ***197*** ***198*** ***562***<br>580 687 723	112 ***113*** ***115*** ***117***<br>***120*** 121 ***122*** ***123***<br>***174*** 176 ***178*** ***179***<br>***180*** ***181*** ***194*** ***196***<br>***197*** ***198*** 200***205*** ***206***<br>***207*** ***209*** 211 ***212***<br>***216*** ***217*** ***224*** ***225***<br>***228*** 229 ***230*** ***562***<br>588	***1*** ***3*** ***4*** ***5*** ***7*** ***12*** ***13*** ***14***<br>16 ***17*** ***18*** ***20*** ***22*** ***23***<br>***25*** ***28*** ***34*** 37 ***38*** ***46***<br>52 ***53*** ***65*** ***78*** ***79*** ***80***<br>***82*** 83 84 85 ***86*** ***88***<br>***92*** ***94*** ***96*** ***97*** ***98*** ***99***<br>101 103 ***113*** ***115***<br>***117*** ***120*** ***122*** ***123***<br>***124*** ***125*** ***126*** ***127***<br>***129*** 131 132 ***143***<br>144 145 ***150*** ***151***<br>***153*** ***155*** ***159*** ***162***<br>***163*** ***166*** ***169*** ***174***

25 S 0.999**	***178 179 180 181***
28 Q 0.994**	***194 196 197 198***
34 H 0.963*	***562***
38 S 0.955*	
46 T 0.996**	
53 Y 0.975*	
65 L 0.968*	
78 L 0.999**	
79 S 0.964*	
80 S 0.992**	
82 A 0.999**	
86 H 0.997**	
88 V 0.968*	
92 L 0.996**	
94 P 0.998**	
96 S 0.980*	
97 C 0.992**	
98 R 0.990**	
99 D 0.996**	
113 R 0.983*	
115 R 0.984*	
117 Q 0.996**	
120 T 0.989*	
122 P 0.983*	
123 S 0.966*	
124 C 0.989*	

125 P 0.998**

126 A 0.996**

127 P 0.960*

129 L 0.998**

143 S 0.988*

150 C 0.995**

151 W 0.993**

153 R 0.991**

155 S 0.997**

159 S 0.994**

162 F 0.974*

163 L 0.995**

166 V 0.993**

169 W 0.969*

174 G 0.996**

178 Y 0.999**

179 G 0.999**

180 D 1.000**

181 L 0.997**

194 C 0.984*

196 G 0.997**

197 C 0.998**

198 S 0.994**

205 E 0.977*

206 G 0.995**

207 V 0.999**

					209 L 0.991**<br>212 L 0.996**<br>216 F 0.989*<br>217 S 1.000**<br>224 Q 0.952*<br>225 D 0.992**<br>228 V 1.000**<br>230 Y 0.999**<br>562 G 0.995**			
NLRC3	-766.71	-765.18	2	0.216		4 41 203	78 79 101 103 157<br>159 161 189	193 195 200
NLRC4	-4490.31	-4486.03	2	0.013*	3 M 0.959*<br>15 V 0.952*<br>17 E 0.963*<br>19 V 0.955*<br>49 F 0.967*<br>52 S 0.973*<br>56 W 0.978*<br>67 Q 0.973*<br>135 S 0.970*<br>614 A 0.954*	***17*** ***19*** ***135*** ***614***	***3*** 4 5 6 7 8 9 10 ***15***<br>***17*** ***19*** ***49*** 51 ***52*** 53<br>54 ***56*** ***67*** 68 283 287<br>307 310 344 347<br>357 358 430 442<br>507 518 599 601<br>604 607 611 ***614***<br>617 621 623 625	***17*** ***19*** 21 23 24 25<br>26 27 28 30 ***49*** ***56***<br>***67*** ***135*** 172 176 364<br>365 ***614*** 681 686<br>713 715
NLRC5	-3264.15	3262.06	2	0.124			1 3 4 9 323	11 14 15 18
NLRP1	-3009.05	-3002.17	2	0.00102*	396 K 0.959*<br>510 M 0.958*	11 14 76 ***396*** ***510***	44 51 76 361 384<br>***396*** 398 399 424<br>425 440 442 445<br>452 469 480	167 179 307 347<br>352 ***396*** ***510***

NLRP2	-1764.48	-1760.12	2	0.012*	197 R 0.993**	***197*** 360	22 110 139 177	***197***
NLRP3	-3704.16	-3704.17	2	0.990			1 920 967 1068	29 232 469 589 678 691 843 918 946 1032
NLRP4	-2969.69	-2969.70	2	0.991		112 132 135 139 359 597	223 256 259	73
NLRP5	-4063.97	-4058.10	2	0.002**		11 14 15	1 2 3 4 76 93 125 129 228 236 279 286	9 323
NLRP6	-3420.51	-3420.530	2	0.980			1 318 342 358	150 156 169 291 403 422 446 451 453 483 497 498 499 580 583
NLRP7	-4515.69	-4514.94	2	0.470		78 149 286	13 26 37 90 106 108 116 176 188 192 198 200 209 214 251 276 288 296 302 332 333 337 340 354 360 365 368 415 448 429 435 870 874 879	405 462 521 661 822 840 847
NLRP8	-2732.38	-2731.62	2	0.469		39 40 352 355 379 383 388	3 4 5 14 18 172 172 198 224 237 257 309 406 432 448 450 460	22 28 36 76 93 110 119 261 265 292 293 183 187 518 519
NLRP9	-4328.05	-4124.60	2	4.39E-89**	2 L 0.966*	***25*** ***26*** ***27*** ***30*** ***32*** ***358***	***2*** ***5*** ***9*** ***25*** ***26*** ***27*** ***30*** ***32***	***10*** ***13*** ***15*** ***17*** 19 ***35***

					5 L 0.979*<br>9 L 0.995**<br>10 P 1.000**<br>13 P 0.997**<br>15 I 0.999**<br>17 L 0.963*<br>25 K 0.996**<br>26 T 0.998**<br>27 F 0.978*<br>30 K 0.994**<br>32 I 0.987*<br>35 L 0.983*<br>38 I 0.985*<br>41 L 0.994**<br>45 A 0.950*<br>46 N 0.999**<br>52 K 0.992**<br>56 K 0.989*<br>57 S 1.000**<br>218 Q 0.985*<br>358 L 0.985*<br>504 H 0.990**		***504***	***38*** ***41*** ***45*** ***46*** ***52*** 54<br>***56*** ***57*** 152 ***218*** 285<br>378 379 380 383<br>393 399 423 473<br>***504***
NLRP10	-4708.12	-4708.08	2	0.964		586 598 632 798<br>912	232 269 418 465<br>555 692 832 849<br>889	15 29 365 388
NLRP11	-7219.09	-7083.12	2	8.86E-60**	129 E 0.987*	***129*** ***289*** ***377*** ***426***	41 128 ***129*** 132 133	179 180 181 184

136 V 0.952*	***427*** ***430***	***136*** ***137*** ***138*** 139	187 ***189*** 190 ***191***
137 K 0.968*		140 ***141*** ***145*** ***146***	193 ***194*** ***198*** ***200***
138 T 0.987*		***147*** ***148*** ***149*** ***150***	***201*** 203 205***206*** ***207***
141 Q 0.993**		151 152 153 ***154***	***210*** ***232*** ***237*** ***241***
145 V 0.968*		***157*** ***160*** ***161*** ***163***	***244*** ***248*** ***250*** ***252***
146 V 0.992**		***164*** ***167*** ***169*** 178	***253*** ***258*** ***262*** ***264***
147 L 0.988*		***206*** ***207*** ***210*** ***212***	***265*** ***267*** ***269*** ***270***
148 Q 0.977*		***214*** ***216*** ***219*** ***221***	***272*** ***275*** ***276*** ***277***
149 G 0.969*		***222*** ***227*** ***230*** ***289***	***278*** ***377*** ***382*** ***386***
150 A 0.986*		***295*** ***297*** ***298*** ***299***	***387*** ***389*** ***418*** 420
154 G 0.994**		***302*** ***303*** ***307*** ***314***	
157 T 0.984*		***316*** ***319*** ***321*** ***323***	
160 R 0.994**		***326*** ***327*** ***330*** 331	
161 K 0.984*		332 333 334 335	
163 L 0.951*		337 338 ***339*** ***340***	
164 L 0.980*		***344*** ***347*** ***352*** ***357***	
167 A 0.979*		359 360 ***362*** 363	
169 G 0.969*		***364*** ***367*** ***370*** ***377***	
189 M 0.990*		***382*** ***386*** ***387*** ***389***	
191 S 0.972*		***403*** ***405*** ***406*** ***411***	
194 W 0.989*		***414*** ***418*** 420 422	
198 E 0.974*		***426*** ***427*** ***430*** ***437***	
200 P 0.993**		438 ***439*** 458 507	
201 V 0.989*		530 555	
206 S 0.991**			
207 Q 0.989*			

210 S 0.960*

212 L 0.988*

214 I 0.992**

216 D 0.969*

219 D 0.958*

221 N 0.993**

222 F 0.990*

227 P 0.962*

230 E 0.985*

232 C 0.978*

237 H 0.987*

241 F 0.950*

244 S 0.990**

248 K 0.979*

250 M 0.986*

252 P 0.961*

253 E 0.982*

258 V 0.987*

262 L 0.979*

264 A 0.950*

265 Y 0.992**

267 K 0.979*

269 K 0.984*

270 P 0.966*

272 L 0.961*

275 Q 0.972*

276 R 0.974*

277 S 0.991**

278 V 0.989*

289 E 0.965*

295 F 0.980*

297 E 0.985*

298 D 0.977*

299 K 0.970*

302 S 0.991**

303 L 0.970*

307 D 0.984*

314 L 0.988*

316 S 0.991**

319 K 0.976*

321 P 0.982*

323 V 0.984*

326 I 0.983*

327 I 0.978*

330 C 0.991**

339 G 0.951*

340 D 0.987*

344 T 0.955*

347 T 0.978*

352 F 0.988*

357 S 0.986*

362 Q 0.973*

					364 D 0.963*			
					367 C 0.989*			
					370 L 0.986*			
					377 R 0.972*			
					382 K 0.983*			
					386 T 0.992**			
					387 M 0.970*			
					389 H 0.978*			
					403 K 0.980*			
					405 D 0.992**			
					406 V 0.985*			
					411 D 0.987*			
					414 I 0.976*			
					418 D 0.981*			
					426 E 0.981*			
					427 F 0.990**			
					430 L 0.988*			
					437 A 0.991**			
					439 M 0.983*			
NLRP12	-2976.43	-2976.44	2	0.990		185 585	411	56 61 519 543
NLRP14	-2972.68	-2972.98	2	0.748			76 79 323 585	126 198 441 498
					1 S 0.970*		***13*** ***15*** 16 ***17*** ***19*** 20	***1*** ***2*** ***3*** ***4*** 5 ***6*** 7 9 10 11
					2 V 0.990**		***21*** ***22*** 24 27 ***31*** ***32***	***13*** ***43*** 44 ***45*** ***47*** ***48***
NLRX1	-2957.14	-2924.98	2	1.08E-14**	3 D 0.973*	***45*** ***47*** ***48*** 109 110	***34*** 35 37 38 ***39*** ***40***	***49*** ***76*** ***79*** 80 81 82
					4 E 0.962*		***43*** 44 ***45*** ***47*** ***48*** 50	***83*** ***87*** ***88*** 89 91 92
					6 I 0.981*		51 ***52*** ***53*** 54 ***55*** 58	***93*** 94 95 96 ***97*** 100

13 I 0.965*<br>15 A 0.964*<br>17 K 0.977*<br>19 A 0.970*<br>21 P 0.993**<br>22 Q 0.999**<br>31 S 0.953*<br>32 G 0.950*<br>34 P 0.973*<br>39 E 0.978*<br>40 Q 0.972*<br>43 R 0.967*<br>45 T 0.954*<br>47 A 0.973*<br>48 T 0.998**<br>49 E 0.979*<br>52 Q 0.970*<br>53 R 0.957*<br>55 R 0.966*<br>60 E 0.964*<br>66 E 0.958*<br>67 E 0.966*<br>70 F 0.960*<br>76 L 0.966*<br>79 V 0.992**<br>83 P 0.956*	59 ***60*** 63 ***66*** ***67*** 68<br>69 ***70*** 71 72 74 112<br>***113*** ***114*** 115 ***116***<br>117 118 ***121*** 123<br>164 ***165*** ***216***	102 103 ***104*** ***105***<br>***107*** ***165*** ***216***

						87 E 0.964*<br>88 S 0.960*<br>93 L 0.968*<br>97 S 0.985*<br>104 Q 0.960*<br>105 L 0.957*<br>107 T 0.997**<br>113 R 0.988*<br>114 R 0.953*<br>116 Q 0.967*<br>121 Y 0.984*<br>165 S 0.996**			
	CIITA	-7816.95	-7812.08	2	0.007**	313 R 0.963*	120 ***313***	70 93 144 1796 182 ***313*** 545	242 248 251
	NAIP	-2136.01	-2137.13	2	0.326		76 198	133 148 149 165 166 167 168 171	22 48 49 52 56 58 168
	NOD1	-9685.35	-9684.76	2	0.558			243 507 509	152 183
Chiroptera	NOD2	-1845.28	-1845.29	2	0.990		121 128 136 309	45 46 48 49 53 56 131 138 139 160 161 169	69 71 78 170 178 201
	NLRC3	-1937.01	-1930.16	2	0.001**	35 R 0.955*	***35*** 327	***35*** 67 69 81 148	***35*** 176
	NLRC4	-8352.37	-8352.29	2	0.923		369 373	18 63 64 66 69 441	72 79 81 86 223 226 229 235
	NLRC5	-11322.86	-11307.13	2	1.43E-07**	158 T 0.962*<br>311 G 0.984*	***158*** ***311*** 404	59 142 ***158*** ***311*** 341 367 369	194 218 234 284 288 ***311*** 416 419 429

NLRP1	-9247.49	-9224.93	2	1.59E-10**	3 A 0.959*<br>4 R 0.991**<br>9 G 0.966*<br>48 R 0.985*<br>53 G 0.981*<br>55 Q 0.952*<br>56 L 0.968*<br>61 C 0.981*<br>98 K 0.953*<br>160 C 0.954*<br>161 G 0.992**<br>169 S 0.961*<br>171 N 0.972*<br>175 E 0.978*<br>209 K 0.976*<br>213 C 0.974*<br>220 D 0.965*	***9*** ***61*** ***98*** ***209***	1 ***3*** ***4*** 8 ***9*** ***48*** 143 144 157 159 ***160*** ***161*** 162 ***169*** ***171*** 505 ***209*** ***213*** ***220*** 221 223 241 247	***48*** 49 ***53*** ***55*** ***56*** ***169*** 170 ***171*** 172 173 174 ***175*** ***209*** 250 267 275 276
NLRP2	-2746.80	-2745.79	2	0.364		19 69	45 46 156 301 302 303	178 189 191 193 195 200 201 206 209 219 265
NLRP3	-7739.74	-7719.30	2	1.33E-09**	141 R 0.970*<br>327 H 0.963*<br>414 Q 0.989*		48 56 66 92 164 264 318 320 323 324 326 386 426	141 327 414
NLRP4	-4821.60	-4818.05	2	0.028*		67	45 49 68 98 131	158 161 162 168 169 325
NLRP5	-12740.27	-12740.03	2	0.786		303 306	63 90 107	265

NLRP6	-3450.29	-3449.45	2	0.431		56 59 321 322 326	123 126 129 439 440 441	243 256
NLRP7	-2883.17	-2882.38	2	0.453		261 282	303	59 63 81 108 193 203 295
NLRP8	-9187.63	-9185.05	2	0.075		85 216 274 275	55 58 62 65 68 233 246 250 257	17 28 36 203 207 208 281 296 312 334
NLRP9	-1835.49	-1835.21	2	0.756			25 32 141 204	73 98 103 199
NLRP10	-9918.38	-9886.33	2	1.24E-14**	481 E 0.967* 489 H 0.998** 508 V 0.988*		***481*** ***489*** ***508*** 513	88 154 211 218 231 289 478 ***481*** ***489*** 496 506
NLRP11	-20760.83	-20910.89	2	6.76E-66**	247 G 0.981*	41 ***247***	115 150 156 159 179 182 186 200 209 219 224 234 238 ***247*** 251 295 296 299 301 304 306 310 319 326	102 104 ***247***
NLRP12	-2550.21	-2550.19	2	0.981		211 269	408	70 88 98 161 202 303 319 347 349 425 465
NLRP13	-5555.16	-5553.28	2	0.153		59 155 180 846	216 218 356 459 469 732 733 756 769	115 145 178 179 180 561 567 589 594
NLRP14	-3477.10	-3477.04	2	0.945			170 176 189	100
NLRX1	-4624.45	-4618.91	2	0.0039**	24 G 0.964* 27 L 0.977*	15 ***24*** ***27*** ***87***	***24*** ***27*** ***87***	***87*** 91 96 132 134 136 256 258 432

	87 L 0.960*	458 489

Notes: * The significant level :*（$0.01 < p < 0.05$）,**（$p < 0.01$）
