# Supplementary material for: Molecular Evolution of the NLR Gene Family Reveals Diverse Innate Immune Strategies in Bats
Source: Biomolecules. 2025 Dec 10;15(12):1715. doi: 10.3390/biom15121715 (PMC12730308; doi:10.3390/biom15121715)
Supplement: Supplementary file 1 [file biomolecules-15-01715-s001.zip › Table S3.pdf]

Table S3. Analysis results of the Chiroptera species Branch Model.

Gene	InL M0	InL M1	p-Values	ω0	ω	Family
CIITA	-7914.07	-7911.14	0.439	0.21334	0.20979	*Vespertilionidae*
					0.21283	*Phyllostomatidae*
					0.37189	*Hipposideridae*
					0.24029	*Emballonuridae*
					0.37293	*Pteropodidae*
					0.20766	*Rhinolophidae*
NAIP	-8621.5	-8626.3	0.137	0.09784	0.27771	*Vespertilionidae*
					0.14710	*Phyllostomatidae*
					0.28053	*Hipposideridae*
					0.10081	*Emballonuridae*
					0.11120	*Pteropodidae*
					0.26629	*Rhinolophidae*
NOD1	-9921.06	-9916.67	0.188	0.12315	0.15228	*Vespertilionidae*
					0.13860	*Phyllostomatidae*
					0.45078	*Hipposideridae*
					0.09546	*Emballonuridae*
					0.13923	*Pteropodidae*
					0.08989	*Rhinolophidae*
NOD2	-1874.07	-1864.73	0.0047**	0.08652	0.25054	*Vespertilionidae*
					0.14534	*Phyllostomatidae*
					0.40715	*Hipposideridae*
					0.06274	*Emballonuridae*
					0.02365	*Pteropodidae*
					0.05068	*Rhinolophidae*
NLRC3	-1956.28	-1951.98	0.197	0.09784	0.10238	*Vespertilionidae*
					0.11536	*Phyllostomatidae*
					0.42669	*Hipposideridae*
					0.15139	*Emballonuridae*
					0.05333	*Pteropodidae*
					0.24146	*Rhinolophidae*
NLRC4	-8339.67	-8341.67	0.135	0.13225	0.23885	*Vespertilionidae*
					0.11435	*Phyllostomatidae*
					0.12442	*Hipposideridae*
					0.10897	*Emballonuridae*
					0.04323	*Pteropodidae*
					0.24211	*Rhinolophidae*
NLRC5	-11498.24	-11491.32	0.031*	0.25957	0.31986	*Vespertilionidae*
					0.20180	*Phyllostomatidae*
					0.35626	*Hipposideridae*
					0.26720	*Emballonuridae*
					0.42560	*Pteropodidae*
					0.18985	*Rhinolophidae*

					1.09167	*Vespertilionidae*
					0.33744	*Phyllostomatidae*
NLRP1	-9314.24	-9302.41	0.0006**	0.78939	0.41384	*Hipposideridae*
					0.36654	*Emballonuridae*
					0.38371	*Pteropodidae*
					0.81333	*Rhinolophidae*
					0.23323	*Vespertilionidae*
					0.26658	*Phyllostomatidae*
NLRP2	-2757.75	-2754.36	0.341	0.26990	0.44950	*Hipposideridae*
					0.15344	*Emballonuridae*
					0.09746	*Pteropodidae*
					0.36400	*Rhinolophidae*
					0.08847	*Vespertilionidae*
					0.07997	*Phyllostomatidae*
NLRP3	-7850.57	-7845.52	0.120	0.10795	0.34404	*Hipposideridae*
					0.17303	*Emballonuridae*
					0.06212	*Pteropodidae*
					0.07770	*Rhinolophidae*
					0.14536	*Vespertilionidae*
					0.43139	*Phyllostomatidae*
NLRP4	-4871.42	-4866.95	0.176	0.19996	0.44175	*Hipposideridae*
					0.03178	*Emballonuridae*
					0.33247	*Pteropodidae*
					0.16431	*Rhinolophidae*
					0.16242	*Vespertilionidae*
					0.57232	*Phyllostomatidae*
NLRP5	-12836.26	-12828.61	0.018*	0.26623	0.38125	*Hipposideridae*
					0.18495	*Emballonuridae*
					0.21061	*Pteropodidae*
					0.34123	*Rhinolophidae*
					0.15851	*Vespertilionidae*
					0.14554	*Phyllostomatidae*
NLRP6	-3453.7	-3451.2	0.082	0.26596	0.14134	*Hipposideridae*
					0.12775	*Emballonuridae*
					0.11343	*Pteropodidae*
					0.13812	*Rhinolophidae*
					0.16369	*Vespertilionidae*
					0.16762	*Phyllostomatidae*
NLRP7	-2897.13	-2894.50	0.511	0.21803	0.40910	*Hipposideridae*
					0.26064	*Emballonuridae*
					0.01644	*Pteropodidae*
					0.29328	*Rhinolophidae*
NLRP8	-9266.23	-9259.60	0.039*	0.36459	0.49167	*Vespertilionidae*
					0.49969	*Phyllostomatidae*

					0.41798	*Hipposideridae*
					0.47073	*Emballonuridae*
					0.39679	*Pteropodidae*
					0.63784	*Rhinolophidae*
					0.14322	*Vespertilionidae*
					0.17339	*Phyllostomatidae*
NLRP9	-2553.25	-2552.36	0.410	0.23128	0.14774	*Hipposideridae*
					0.09875	*Emballonuridae*
					0.16338	*Pteropodidae*
					0.10219	*Rhinolophidae*
					0.26962	*Vespertilionidae*
					0.42697	*Phyllostomatidae*
NLRP10	-10149.93	-10143.73	0.053	0.33784	0.34295	*Hipposideridae*
					0.24696	*Emballonuridae*
					0.31258	*Pteropodidae*
					0.50141	*Rhinolophidae*
					0.25650	*Vespertilionidae*
					0.71437	*Phyllostomatidae*
NLRP11	-20802.20	-20792.76	0.0043**	0.33215	0.35365	*Hipposideridae*
					0.00010	*Emballonuridae*
					0.38437	*Pteropodidae*
					0.13866	*Rhinolophidae*
					0.13116	*Vespertilionidae*
					0.12339	*Phyllostomatidae*
NLRP12	-2581.75	-2578.87	0.451	0.08941	0.35461	*Hipposideridae*
					0.10626	*Emballonuridae*
					0.05753	*Pteropodidae*
					0.03858	*Rhinolophidae*
					0.36711	*Vespertilionidae*
					0.47657	*Phyllostomatidae*
NLRP13	-5612.69	-5607.18	0.087	0.34568	0.43385	*Hipposideridae*
					0.43236	*Emballonuridae*
					0.43863	*Pteropodidae*
					0.53263	*Rhinolophidae*
					0.10121	*Vespertilionidae*
					0.38157	*Phyllostomatidae*
NLRP14	-3510.21	-3504.17	0.061	0.24163	0.35213	*Hipposideridae*
					0.20526	*Emballonuridae*
					0.20324	*Pteropodidae*
					0.38662	*Rhinolophidae*
					0.35173	*Vespertilionidae*
					0.29354	*Phyllostomatidae*
NLRX1	-4660.98	-4650.68	0.0021**	0.25825	0.33764	*Hipposideridae*
					0.24836	*Emballonuridae*

0.45889	*Pteropodidae*
0.48704	*Rhinolophidae*

Notes: * The significant level :*（$0.01 < p < 0.05$）,**（$p < 0.01$）
