# Supplementary material for: Molecular Evolution of the NLR Gene Family Reveals Diverse Innate Immune Strategies in Bats
Source: Biomolecules. 2025 Dec 10;15(12):1715. doi: 10.3390/biom15121715 (PMC12730308; doi:10.3390/biom15121715)
Supplement: Supplementary file 1 [file biomolecules-15-01715-s001.zip › Table S4.pdf]

Table S4. Positive selection for NLR gene branch locus models in Chiroptera.

Family	Gene	lnL M1a	lnL M2a	LRT	df	*P*-values	BEB
Vespertilionidae	*CIITA*	-7825.50	-7825.50	0	1	1	
	NAIP	-4329.37	-4329.37	0	1	1	
	NLRC3	-1932.01	-1932.01	0	1	1	
	NLRC4	-5138.73	-5137.91	1.64	1	0.200	
	NLRC5	-11271.59	-11272.59	2	1	0.157	
	NLRP1	-9188.81	-9235.35	93.08	1	5.02E-22**	4 R 0.999**<br>6 G 0.978*<br>7 L 0.986*<br>13 K 0.966*<br>14 A 0.972*<br>17 C 0.981*<br>20 R 0.971*<br>21 I 0.978*<br>22 S 0.995**<br>23 P 1.000**<br>24 D 0.998**<br>25 L 0.980*<br>30 L 0.991**<br>31 S 0.996**<br>36 R 0.962*<br>38 A 0.976*<br>39 G 0.969*<br>40 A 0.970*<br>41 Q 0.992**<br>48 R 1.000**<br>49 H 0.958*<br>51 S 0.999**<br>53 G 0.992**<br>55 Q 0.999**<br>59 V 0.962*<br>60 G 0.999**<br>63 L 0.991**<br>64 T 0.956*<br>68 S 0.992**<br>73 N 0.979*<br>75 I 0.988*<br>76 L 0.963*<br>77 S 0.988*<br>81 L 0.999**<br>82 R 0.992**
	NLRP2	-2748.95	-2749.01	0.12	1	0.729	
	NLRP3	-7740.93	-7740.93	0	1	1	

	NLRP4	-4831.06	-4831.56	1	1	0.317	
	NLRP5	-12771.70	-12771.70	0	1	1	
	NLRP6	-7241.63	-7241.63	0	1	1	
	NLRP7	-2888.27	-2888.35	0.16	1	0.689	
	NLRP8	-9217.99	-9195.06	45.86	1	1.27E-11**	25 P 0.954*<br>86 S 0.972*<br>251 G 0.959*<br>266 L 0.950*<br>290 R 0.961*<br>292 G 0.952*<br>324 C 0.955*
	NLRP9	-2765.38	-2765.38	0	1	1	
	NLRP10	-9925.75	-9925.75	0	1	1	
	NLRP11	-20910.89	-20910.89	0	1	1	
	NLRP12	-2571.26	-2571.27	0.02	1	0.888	
	NLRP13	-5556.23	-5556.23	0	1	1	
	NLRP14	-3487.08	-3487.08	0	1	1	
	NLRX1	-4614.03	-4614.03	0	1	1	
	NOD1	-9757.63	-9757.63	0	1	1	
	NOD2	-1856.91	-1856.91	0	1	1	
	CIITA	-7825.50	-7825.50	0	1	1	
	NAIP	-4329.37	-4329.37	0	1	1	
	NLRC3	-1932.01	-1932.01	0	1	1	
	NLRC4	-5138.62	-5138.59	0.06	1	0.806	
	NLRC5	-11336.66	-11336.66	0	1	1	
	NLRP1	-9231.51	-9231.51	0	1	1	
	NLRP2	-2748.54	-2748.82	0.56	1	0.454	
	NLRP3	-7740.93	-7740.93	0	1	1	
	NLRP4	-4832.08	-4832.08	0	1	1	
	NLRP5	-12775.43	-12776.39	1.92	1	0.166	
Phyllostomatidae	*NLRP6*	-7241.63	-7241.63	0	1	1	
	NLRP7	-2888.93	-2888.93	0	1	1	
	NLRP8	-9195.06	-9195.06	0	1	1	
	NLRP9	-2766.17	-2766.17	0	1	1	
	NLRP10	-9923.97	-9923.97	0	1	1	
	NLRP11	-20910.89	-20910.89	0	1	1	
	NLRP12	-2572.83	-2572.83	0	1	1	
	NLRP13	-5555.91	-5556.12	0.42	1	0.517	
	NLRP14	-3488.97	-3488.97	0	1	1	
	NLRX1	-4622.54	-4622.54	0	1	1	
	NOD1	-9754.76	-9757.68	5.84	1	0.016*	443 A 0.995**
	NOD2	-1864.05	-1864.05	0	1	1	
Hipposideridae	*CIITA*	-7825.50	-7825.49	0.02	1	0.888	
	NAIP	-4329.19	-4328.67	1.04	1	0.308	

NLRC3	-1930.33	-1930.33	0	1	1	
NLRC4	-5137.66	-5137.66	0	1	1	
NLRC5	-11336.66	-11336.66	0	1	1	
NLRP1	-9235.35	-9231.20	8.3	1	0.004**	
NLRP2	-2746.11	-2749.24	6.26	1	0.012*	8 L 0.969*
NLRP3	-7725.43	-7725.43	0	1	1	
NLRP4	-4831.06	-4832.91	3.7	1	0.054	
NLRP5	-12776.06	-12776.06	0	1	1	
NLRP6	-7241.63	-7241.63	0	1	1	
NLRP7	-2888.73	-2888.88	0.3	1	0.584	
NLRP8	-9195.06	-9195.06	0	1	1	
NLRP9	-2766.17	-2766.17	0	1	1	
NLRP10	-9917.88	-9923.28	10.8	1	0.001**	296 E 0.983*<br>472 Q 0.972*
NLRP11	-20910.89	-20910.89	0	1	1	
NLRP12	-2572.94	-2572.94	0	1	1	
NLRP13	-5555.17	-5556.27	2.2	1	0.138	
NLRP14	-3490.33	-3490.33	0	1	1	
NLRX1	-4544.14	-4605.72	123.16	1	1.29E-28**	1 I 1.000**<br>4 G 0.961*<br>7 R 0.994**<br>8 H 0.997**<br>9 P 0.961*<br>10 D 0.995**<br>11 E 0.998**<br>14 D 0.983*<br>15 E 0.994**<br>16 V 1.000**<br>17 F 1.000**<br>18 E 1.000**<br>19 L 0.994**<br>20 P 1.000**<br>21 M 1.000**<br>22 F 0.994**<br>23 M 0.999**<br>24 G 0.990*<br>26 L 0.997**<br>27 L 0.991**<br>28 S 1.000**<br>29 A 0.995**<br>32 M 0.999**<br>33 L 0.986*<br>34 A 0.999**<br>35 Q 1.000**

							36 L 0.982*<br>37 G 1.000**<br>39 P 1.000**<br>40 I 1.000**<br>41 R 1.000**<br>42 N 1.000**<br>43 L 1.000**<br>44 D 0.987*<br>45 A 1.000**<br>46 L 0.999**<br>47 E 0.986*<br>48 N 1.000**<br>49 A 0.984*<br>50 Q 0.993**
	NOD1	-9761.50	-9761.50	0	1	1	
	NOD2	-1863.48	-1863.48	0	1	1	
	CIITA	-7823.03	-7825.43	4.8	1	0.028	
	NAIP	-4329.53	-4330.1	1.14	1	0.286	
Emballonuridae	*NLRC3*	-1941.39	-1935.23	12.32	1	0.00045**	1 L 0.959*<br>3 R 0.986*<br>4 L 0.996**<br>9 S 0.996**<br>12 S 0.973*<br>15 P 0.993**<br>18 S 0.993**<br>20 T 0.997**<br>32 L 0.955*<br>37 Q 0.995**<br>48 T 0.994**<br>49 H 0.975*<br>52 L 0.993**<br>53 S 0.993**<br>55 D 0.987*<br>62 F 0.984*<br>66 A 0.994**<br>73 I 0.981*<br>74 L 0.993**<br>78 D 0.990*<br>80 C 0.966*<br>87 N 0.987*<br>91 C 0.993**<br>93 D 0.958*<br>95 K 0.983*<br>97 E 0.960*

							99 Q 0.969*<br>101 D 0.993**<br>103 L 0.994**<br>104 I 0.987*
	NLRC4	-5137.91	-5137.91	0	1	1	
	NLRC5	-11336.66	-11336.66	0	1	1	
	NLRP1	-9231.51	-9231.51	0	1	1	
	NLRP2	-2749.24	-2749.24	0	1	1	
	NLRP3	-7740.49	-7740.53	0.08	1	0.777	
	NLRP4	-4833.10	-4833.10	1	1	1	
	NLRP5	-12777.59	-12777.59	0	1	1	
	NLRP6	-7241.63	-7241.63	0	1	1	
	NLRP7	-2888.91	-2888.91	0	1	1	
	NLRP8	-9194.53	-9194.40	0.26	1	0.610	
	NLRP9	-2767.56	-2767.56	0	1	1	
	NLRP10	-9917.20	-9920.71	7.02	1	0.008**	114 D 0.990*<br>128 V 0.991**
	NLRP11	-20910.89	-20910.89	0	1	1	
	NLRP12	-2573.02	-2573.13	0.22	1	0.639	
	NLRP13	-5552.46	-5552.46	0	1	1	
	NLRP14	-3490.33	-3490.33	0	1	1	
	NLRX1	-4622.54	-4622.53	0.02	1	0.888	
	NOD1	-9761.50	-9761.50	0	1	1	
	NOD2	-1864.05	-1864.05	0	1	1	
	CIITA	-7823.43	-7824.79	2.72	1	0.099	
	NAIP	-4330.82	-4330.82	0	1	1	
	NLRC3	-1932.01	-1932.01	0	1	1	
	NLRC4	-5137.91	-5137.91	0	1	1	
	NLRC5	-11336.66	-11336.66	0	1	1	
	NLRP1	-9231.51	-9231.51	0	1	1	
	NLRP2	-2749.24	-2749.24	0	1	1	
	NLRP3	-7740.93	-7740.93	0	1	1	
	NLRP4	-4831.43	-4831.43	0	1	1	
Pteropodidae	*NLRP5*	-12777.59	-12777.59	0	1	1	
	NLRP6	-7241.63	-7241.63	0	1	1	
	NLRP7	-2888.57	-2888.62	0.1	1	0.752	
	NLRP8	-9195.06	-9195.06	0	1	1	
	NLRP9	-2766.17	-2766.17	0	1	1	
	NLRP10	-9924.02	-9924.19	0.34	1	0.560	
	NLRP11	-20910.89	-20910.89	0	1	1	
	NLRP12	-2573.13	-2573.13	0	1	1	
	NLRP13	-5552.46	-5552.46	0	1	1	
	NLRP14	-3489.92	-3490.33	0.82	1	0.365	
	NLRX1	-4622.54	-4622.10	0.88	1	0.348	

	NOD1	-9760.62	-9760.62	0	1	1	
	NOD2	-1864.05	-1864.05	0	1	1	
	CIITA	-7824.68	-7824.68	0	1	1	
	NAIP	-4330.82	-4330.82	0	1	1	
	NLRC3	-1932.01	-1930.81	2.4	1	0.121	
	NLRC4	-5138.63	-5138.63	0	1	1	
	NLRC5	-11336.66	-11336.66	0	1	1	
	NLRP1	-9213.15	-9227.3	28.3	1	1.04E-07**	192 L 0.986*<br>208 C 0.999**<br>213 C 0.978*<br>253 G 0.991**<br>276 L 0.999**
	NLRP2	-2749.17	-2749.17	0	1	1	
	NLRP3	-7732.62	-7732.62	0	1	1	
	NLRP4	-4833.10	-4832.10	2	1	0.157	
	NLRP5	-12775.07	-12775.07	0	1	1	
	NLRP6	-7241.63	-7241.63	0	1	1	
	NLRP7	-2888.39	-2888.52	0.26	1	0.610	
	NLRP8	-9195.06	-9195.06	0	1	1	
	NLRP9	-2765.38	-2765.38	0	1	1	
Rhinolophidae	*NLRP10*	-9901.91	-9923.13	42.44	1	7.29E-11**	1 S 1.000**
	NLRP11	-20910.89	-20910.89	0	1	1	
	NLRP12	-2573.13	-2573.13	0	1	1	
	NLRP13	-5548.45	-5553.50	10.1	1	0.001**	189 A 0.992**
	NLRP14	-3490.33	-3490.33	0	1	1	
	NLRX1	-4599.95	-4609.71	19.52	1	9.96E-06**	1 I 0.957*<br>16 V 0.952*<br>17 F 0.973*<br>21 M 0.974*<br>22 F 0.952*<br>28 S 0.972*<br>34 A 0.975*<br>35 Q 0.973*<br>42 N 0.951*<br>43 L 0.960*<br>48 N 0.987*
	NOD1	-9761.41	-9761.41	0	1	1	
	NOD2	-1864.05	-1864.05	0	1	1	

Notes: * The significant level :*（$0.01 < p < 0.05$）,**（$p < 0.01$）
