# Supplementary material for: Molecular Evolution of the NLR Gene Family Reveals Diverse Innate Immune Strategies in Bats
Source: Biomolecules. 2025 Dec 10;15(12):1715. doi: 10.3390/biom15121715 (PMC12730308; doi:10.3390/biom15121715)
Supplement: Supplementary file 1 [file biomolecules-15-01715-s001.zip › Table S5.pdf]

Table S5. The location of positively selected sites within functional domains and their corresponding functional implications.

Gene	Identity with sites of Rhinolophus ferrumequinum	Function information
CIITA	360R	transcriptional regulator ICP4
NLRC3	57R	--
NLRC5	169 T	--
NLRC5	322L	NACHT domain<br>Signal transduction mechanisms
NLRP1	208A	
NLRP1	209G	
NLRP1	253R	
NLRP1	258K	apoptosis proteins and MHC transcription activation
NLRP1	260C	
NLRP1	261A	
NLRP1	271L	
NLRP1	365Y	
NLRP1	366F	--
NLRP1	374R	--
NLRP1	376F	--
NLRP1	380E	--
NLRP1	414L	--

NLRP1	418T	--
NLRP1	425H	--
NLRP3	160R	apoptosis proteins and MHC transcription activation
NLRP3	346W	--
NLRP3	433N	--
NLRP10	490K	helical domain HD2
NLRP10	509S	helical domain HD2
NLRP10	789E	--
NLRP11	769G	TLRR, increasing PP1's activity
NLRX1	38G	
NLRX1	41T	Signal transduction mechanisms
NLRX1	101P	
